# Microvascular resistance of the culprit coronary artery in acute ST-elevation myocardial infarction

**DOI:** 10.1172/jci.insight.85768

**Published:** 2016-05-05

**Authors:** David Carrick, Caroline Haig, Jaclyn Carberry, Vannesa Teng Yue May, Peter McCartney, Paul Welsh, Nadeem Ahmed, Margaret McEntegart, Mark C. Petrie, Hany Eteiba, Mitchell Lindsay, Stuart Hood, Stuart Watkins, Ahmed Mahrous, Samuli M.O. Rauhalammi, Ify Mordi, Ian Ford, Aleksandra Radjenovic, Naveed Sattar, Keith G. Oldroyd, Colin Berry

**Affiliations:** 1 BHF Glasgow Cardiovascular Research Centre, Institute of Cardiovascular and Medical Sciences, University of Glasgow, Glasgow, United Kingdom.; 2 West of Scotland Heart and Lung Centre, Golden Jubilee National Hospital, Clydebank, United Kingdom.; 3 Robertson Centre for Biostatistics, University of Glasgow, Glasgow, United Kingdom.

## Abstract

**BACKGROUND.** Failed myocardial reperfusion is common and prognostically important after acute ST-elevation myocardial infarction (STEMI). The purpose of this study was to investigate coronary flow reserve (CFR), a measure of vasodilator capacity, and the index of microvascular resistance (IMR; mmHg × s) in the culprit artery of STEMI survivors.

**METHODS.** IMR (*n* = 288) and CFR (*n* = 283; mean age [SD], 60 [12] years) were measured acutely using guide wire–based thermodilution. Cardiac MRI disclosed left ventricular pathology, function, and volumes at 2 days (*n* = 281) and 6 months after STEMI (*n* = 264). All-cause death or first heart failure hospitalization was independently adjudicated (median follow-up 845 days).

**RESULTS.** Myocardial hemorrhage and microvascular obstruction occurred in 89 (42%) and 114 (54%) patients with evaluable T2*-MRI maps. IMR and CFR were associated with microvascular pathology (none vs. microvascular obstruction only vs. microvascular obstruction and myocardial hemorrhage) (median [interquartile range], IMR: 17 [12.0–33.0] vs. 17 [13.0–39.0] vs. 37 [21.0–63.0], *P* < 0.001; CFR: 1.7 [1.4–2.5] vs. 1.5 [1.1–1.8] vs. 1.4 [1.0–1.8], *P* < 0.001), whereas thrombolysis in myocardial infarction blush grade was not. IMR was a multivariable associate of changes in left ventricular end-diastolic volume (regression coefficient [95% CI] 0.13 [0.01, 0.24]; *P* = 0.036), whereas CFR was not (*P* = 0.160). IMR (5 units) was a multivariable associate of all-cause death or heart failure hospitalization (*n* = 30 events; hazard ratio [95% CI], 1.09 [1.04, 1.14]; *P* < 0.001), whereas CFR (*P* = 0.124) and thrombolysis in myocardial infarction blush grade (*P* = 0.613) were not. IMR had similar prognostic value for these outcomes as <50% ST-segment resolution on the ECG.

**CONCLUSIONS.** IMR is more closely associated with microvascular pathology, left ventricular remodeling, and health outcomes than the angiogram or CFR.

**TRIAL REGISTRATION.** NCT02072850.

**FUNDING.** A British Heart Foundation Project Grant (PG/11/2/28474), the National Health Service, the Chief Scientist Office, a Scottish Funding Council Senior Fellowship, a British Heart Foundation Intermediate Fellowship (FS/12/62/29889), and a nonfinancial research agreement with Siemens Healthcare.

## Abstract 2

The index of microvascular resistance correlates with microvascular pathology and is a diagnostic test of myocardial reperfusion in patients with acute ST-elevation myocardial infarction.

## Introduction

Acute ST-elevation myocardial infarction (STEMI) is a major cause of premature death and heart failure acutely and in the longer term (1, 2). Primary percutaneous coronary intervention (PCI), the evidence-based standard of care (1, 3), aims to rapidly restore blood flow in the culprit coronary artery by balloon angioplasty followed by stent implantation. The success of this intervention, defined as normal or near normal coronary blood flow, as indicated by thrombolysis in myocardial infarction (TIMI) flow grades 2 and 3, typically occurs in ≥90% of patients (4, 5). Despite routine successful coronary reperfusion, a failure of myocardial reperfusion, manifesting initially as microvascular obstruction within the infarct core and subsequently by vascular degradation and hemorrhagic transformation (6), typically affects half of all patients with acute STEMI and is an adverse prognostic complication (7–9).

The improved early survival rates following acute STEMI in the past decade have been associated with a persistently high incidence of heart failure in the long term (10). One explanation for this conundrum is that more people are surviving the incident STEMI event, but with injured hearts that increase the risk of future heart failure. Specifically, the failure to reperfuse the myocardium, and the related pathologic sequelae of microvascular obstruction and myocardial hemorrhage, are causally implicated in the development of adverse left ventricular (LV) remodeling, heart failure, and death (5, 11–13).

Cardiac MRI is the reference diagnostic test for detection of microvascular pathology; however, MRI is not routinely performed in clinical practice (1, 3). The ECG is the standard-of-care test for failed myocardial reperfusion (1); however, the surface ECG has limited sensitivity for microvascular obstruction (14). The TIMI myocardial perfusion grade is an angiographic method to describe the filling and clearance of radiographic contrast in the myocardium, but it is mainly used for research (15, 16). Accordingly, an invasive diagnostic test that could be routinely used to assess for failed myocardial reperfusion (that is commonly missed) would potentially represent a practice advance.

Coronary guide wire–based sensor technologies have emerged as new diagnostic tools for the invasive management of coronary artery disease (3). Coronary flow reserve (CFR) reflects epicardial and microvascular vasodilator capacity (17, 18). The index of microvascular resistance (IMR) is a direct measure of coronary microvascular resistance. IMR is the product of distal coronary pressure and the mean transit time(s) of a 3 ml intracoronary bolus of saline, manually administered at room temperature, during hyperemia, achieved with i.v. adenosine (140 μg/kg/min) (19). IMR has superior reproducibility and less hemodynamic dependence than CFR (20) but is not independent of LV mass, unlike CFR (21).

The comparative pathophysiological and prognostic significance of IMR and CFR in patients with acute STEMI is incompletely understood (22–24). Since IMR and CFR reflect microvascular resistance and vascular reactivity, respectively, their associations with microvascular pathology within the infarct zone, as revealed noninvasively by MRI, and systemic immune activation, as revealed by circulating concentrations of the cytokine IL-6 (25, 26) and C-reactive protein (CRP), may differ. IMR and CFR may not be interchangeable, but instead each metric may reflect different states of acute vascular injury (27).

We aimed to measure IMR and CFR in the culprit coronary artery immediately after emergency PCI in a large, relatively unselected population of patients with acute STEMI enrolled during daily practice. The specific aims were to assess (a) the feasibility of routine guide wire–based assessments of microvascular function during routine care; (b) the relationships of IMR and CFR with infarct pathologies, including myocardial hemorrhage and microvascular obstruction, revealed by cardiac MRI; (c) the relationships of IMR and CFR with circulating concentrations of IL-6; and (d) the prognostic relationships of IMR and CFR with prespecified LV surrogate outcomes, as revealed by MRI and N-terminal pro b-type natriuretic peptide (NT-proBNP) concentration at 6 months and all-cause death and heart failure hospitalization and major adverse cardiac events (MACE) in the longer term.

We hypothesized that (a) CFR would be more closely associated with microvascular obstruction, a potentially reversible pathology, whereas IMR would be more closely associated with irreversible microvascular damage, as revealed by myocardial hemorrhage; (b) IMR and CFR would be more closely associated with microvascular pathology, as revealed by MRI, than established measures of failed myocardial reperfusion, including the duration of ischaemia, the persistent ST-segment elevation on the surface ECG, and angiographic parameters; and (c) IMR has greatest prognostic significance based on its association with irreversible vascular damage, surrogate outcomes, and all-cause death and heart failure.

### Statistics.

Categorical variables are expressed as number and percentage of patients. Most continuous variables followed a normal distribution and are therefore presented as means together with SD. Those variables that did not follow a normal distribution are presented as medians with interquartile range (IQR). Differences between independent groups were assessed using 1-way ANOVA, Kruskal-Wallis tests, or Fisher’s tests where appropriate. Univariable and multivariable logistic regression analyses were performed to identify predictors of intramyocardial hemorrhage and microvascular obstruction. Models were compared using Harrel’s C-statistic. Kaplan-Meier and Cox proportional hazards methods were used to identify potential predictors of (a) all-cause death and heart failure events and (b) MACE. The assumption of proportional hazards was assessed using Schoenfield residuals. All statistical analyses were carried out using R v 2.15.1 or later. A *P* value of greater than 0.05 indicates the absence of evidence for a statistically significant effect.

## Results

### Patient characteristics and culprit CFR and IMR following coronary reperfusion.

**Figure 1:**
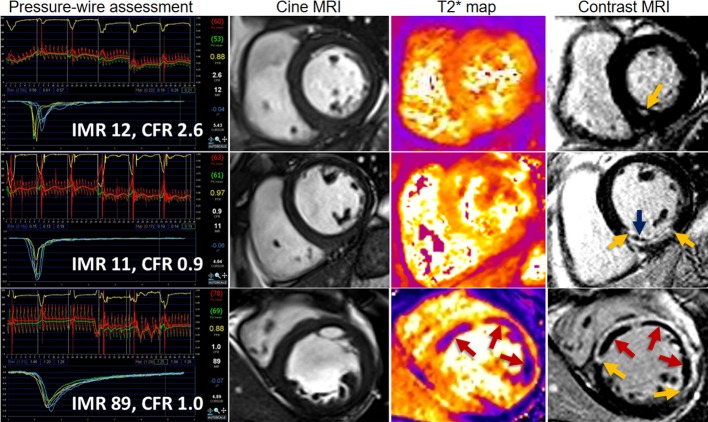
Three patients with acute ST-elevation myocardial infarction treated by primary percutaneous coronary intervention and with the same antithrombotic therapies, including aspirin, clopidogrel, heparin, and glycoprotein IIbIIIa inhibitor therapy with tirofiban. Each patient had successful primary percutaneous coronary intervention (PCI), as evidenced by normal thrombolysis in myocardial infarction (TIMI) flow grade 3 the end of the procedure. Cardiac MRI was performed for each patient 2 days later. Coronary artery function was measured in 283 patients, of whom 281 (99%) had cardiac MRI and 213 (75%) had T2* MRI for assessment of myocardial hemorrhage. Top: A patient with normal index of microvascular resistance (IMR <25), normal coronary flow reserve (CFR >2.0), and no evidence of microvascular injury on MRI. A diagnostic guide wire study of microvascular function in the territory of the culprit coronary artery immediately after primary PCI. IMR and CFR measurements were derived from coronary thermodilution. Microvascular function was normal (IMR 12, CFR 2.6), indicating successful myocardial reperfusion. Two days later, MRI ruled out myocardial hemorrhage (middle right image) or microvascular obstruction (right). Middle: A patient with normal IMR, low CFR, and microvascular obstruction but no hemorrhage on MRI 2 days later. The diagnostic guide wire study of culprit artery microvascular function at the end of primary PCI indicated an abnormal CFR (0.9) but a preserved IMR (11). Late gadolinium contrast-enhanced MRI revealed microvascular obstruction (right image, blue arrow). Bottom: A patient with high IMR, low CFR, and hemorrhagic infarction on MRI. The diagnostic guide wire study of culprit microvascular function immediately after primary PCI indicated severe microcirculatory dysfunction (IMR 89, CFR 1.0). T2*-MRI (middle right image) revealed myocardial hemorrhage (red arrow) within the infarct core. Contrast-enhanced MRI revealed microvascular obstruction (right image, red arrow) within the bright area of infarction. The microvascular obstruction within the infarct core spatially corresponded with the myocardial hemorrhage.

**Figure 2:**
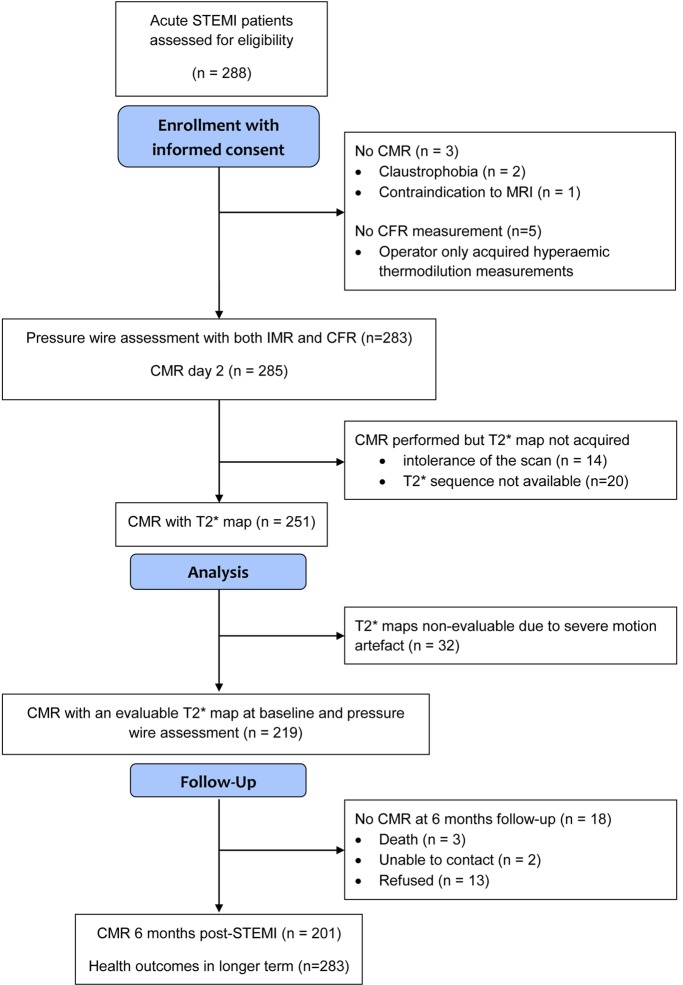
CONSORT flow diagram of the cohort study. STEMI, ST-elevation myocardial infarction; CMR, cardiac magnetic resonance; CFR, coronary flow reserve; IMR, index of microvascular resistance.

283 STEMI patients had IMR (Table 1 and Supplemental Table 1; supplemental material available online with this article; doi:10.1172/jci.insight.85768DS1) and CFR (Supplemental Table 2) measured in the culprit coronary artery without complication at the end of emergency PCI followed by cardiac MRI 2.1 ± 1.8 days later. Case examples are shown in Figure 1. 264 (93%) patients had a second MRI scan to assess LV outcomes 6 months later (Figure 2).

The median IMR and CFR were 25 and 1.6, respectively, with IQRs of 15–48 and 1.1–2.1. An increased IMR (an IMR > median), a reduced CFR (a CFR < median), or both occurred in 136 (48%), 205 (72%), and 107 (38%) patients, respectively.

### Myocardial hemorrhage and microvascular obstruction disclosed by cardiac MRI 2 days after reperfusion.

The MRI findings are described in Table 2 and Supplemental Table 3, respectively. Intraobserver and interobserver reliability for myocardial hemorrhage is described in the Supplemental Results.

213 (97%) patients had T2*-MRI maps and IMR and CFR measurements (Table 3). Myocardial hemorrhage and microvascular obstruction occurred in 89 (42%) and 114 (54%) patients 2 days after reperfusion, respectively.

### Relationships between IMR after reperfusion and incident infarct pathologies 2 days later.

**Figure 3:**
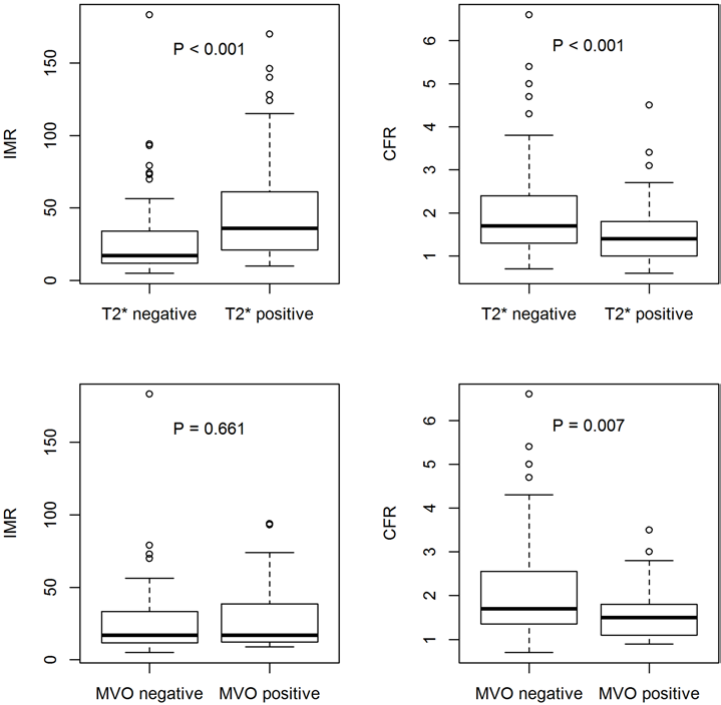
An index of microvascular resistance and coronary flow reserve according to the presence or absence of myocardial hemorrhage and microvascular obstruction. Top: An index of microvascular resistance (IMR) and coronary flow reserve (CFR) according to the presence (*n* = 89 [42%]) or absence (*n* = 124 [58%]) of myocardial hemorrhage in 213 participants who had T2* mapping with MRI 2 days after reperfusion. IMR was higher and CFR was lower in patients with myocardial hemorrhage (T2*MRI positive) compared to that in patients without myocardial hemorrhage (T2* MRI negative). Bottom: IMR and CFR according to the presence (*n* = 25) or absence of microvascular obstruction (*n* = 99) in the subset of patients from above without myocardial hemorrhage (*n* = 124 [58%]). In this subset of patients with less severe vascular injury, IMR was similar in patients with or without microvascular obstruction (MVO), as revealed by contrast-enhanced MRI. By contrast, CFR was lower in patients with MVO compared to CFR in patients without MVO. Mann-Whitney tests were used for the statistical analysis. In box-and-whisker plots, horizontal bars indicate the medians, boxes indicate 25th to 75th percentiles, and whiskers indicate 10th and 90th percentiles.

IMR was higher in patients with myocardial hemorrhage (median [IQR], 37 [21–63]) than in patients without myocardial hemorrhage (median [IQR], 17 [12–33]), including those patients that had microvascular obstruction in the absence of myocardial hemorrhage (median [IQR], 17 [13–39]; *P* < 0.001) (Figure 3 and Supplemental Table 3). IMR was more strongly associated with myocardial hemorrhage (odds ratio [95% CI], 4.24 [2.38, 7.58]; *P* < 0.001) than with microvascular obstruction (odds ratio [95% CI], 2.84 (1.70, 4.73); *P* < 0.001).

The optimal cut-offs for IMR in predicting myocardial hemorrhage and microvascular obstruction are summarized in Supplemental Table 4. Considering myocardial hemorrhage, an IMR >27 had a negative predictive value of 0.74 (0.65, 0.82).

### Relationships between CFR after reperfusion and incident microvascular pathology 2 days later.

CFR was lower in patients with myocardial hemorrhage (odds ratio [95% CI], 1.4 [1.0–1.8]) compared with patients without myocardial hemorrhage (1.7 [1.4–2.5]), including the subset of patients that had microvascular obstruction in the absence of myocardial hemorrhage (1.5 [1.1–1.8]) (*P* < 0.001) (Figure 3).

### Relationships between ECG evidence of failed myocardial reperfusion and incident microvascular pathology 2 days later.

Seventy-eight (28%) patients had persistent ST-segment elevation defined as <50% resolution from the prereperfusion ECG 60 minutes after reperfusion. Of these patients, myocardial hemorrhage occurred in 38 (sensitivity 0.61, specificity 0.66, positive predictive value 0.43, negative predictive value 0.80) and microvascular obstruction occurred in 54 (sensitivity 0.69, specificity 0.57, positive predictive value 0.38, negative predictive value 0.83).

Forty (14%) patients had persistent ST-segment elevation defined as <30% resolution from the prereperfusion ECG 60 minutes after reperfusion. Of these patients, myocardial hemorrhage occurred in 17 (sensitivity 0.19, specificity 0.89, positive predictive value 0.55, negative predictive value 0.60) and microvascular obstruction occurred in 26 (sensitivity 0.18, specificity 0.90, positive predictive value 0.65, negative predictive value 0.52).

### Multivariable associations among IMR, CFR and microvascular pathologies.

IMR (Tables 4 and 5) and CFR (Supplemental Tables 5 and 6) were multivariable associates of microvascular obstruction and myocardial hemorrhage, respectively.

### Associations of IMR and CFR after reperfusion with the circulating acute-phase response.

Blood samples had been collected in the subset of STEMI patients who had been enrolled during office hours. IL-6, which is an immune cytokine that reflects endothelial activation, was measured in 121 and 151 patients 1 day and 6 months after STEMI, respectively. The clinical characteristics of these patients, and the associations with IMR and CFR, were similar to those of the main study population (Supplemental Tables 7 and 8).

IMR was positively associated with IL-6. The IMR values in groups of patients categorized by tertiles of IL-6 concentration at baseline were 19 (IQR, 15–34) versus 23 (IQR, 16–34) versus 30 (IQR, 20–60) (*P* = 0.008). IMR was associated with log IL-6 at baseline (regression coefficient [95% CI], 0.07 [0.04, 0.10]; *P* < 0.001] and the within-subject change in log IL-6 at 6 months from baseline (regression coefficient [95% CI], 0.23 [0.0, 0.10]; *P* < 0.001).

CFR measured acutely was inversely associated with IL-6 concentration on day 1. The CFR values in groups of patients categorized by tertiles of IL-6 (<5.4 pg/ml vs. 5.4 to ≤8.9 pg/ml vs. >8.9 pg/ml) were 1.6 (IQR, 1.3, 2.2) versus 1.5 (IQR, 1.2, 2.1) versus 1.2 (IQR, 1.0, 1.7), respectively (*P* = 0.028). CFR was not associated with log IL-6 at baseline or the within-subject change in IL-6 (data not shown).

In order to investigate the relationships between IMR and IL-6 and the severity of microvascular pathology, as revealed by cardiac MRI, patients were categorized for the occurrence of microvascular obstruction or myocardial hemorrhage. In patients with myocardial hemorrhage (*n* = 41), IMR was associated with the natural log of IL-6 concentration at baseline (regression coefficient [95% CI], 0.11 [0.06, 0.16]; *P* < 0.001) and the within-subject change in log IL-6 at 6 months from baseline (*n* = 25) (regression coefficient [95% CI], –2.94 [–5.01, –0.87]; *P* = 0.008), whereas no associations were observed in patients without myocardial hemorrhage (*n* = 59), including in the subgroup of patients (*n* = 9) with microvascular obstruction.

### Associations for IMR and CFR with NT-proBNP, a biochemical measure of LV remodeling.

NT-proBNP results were available in 121 patients at baseline (index admission) and 152 patients at 6-month follow-up. IMR was associated with NT-proBNP at baseline (regression coefficient [95% CI] 9.2 [4.07, 21.3]; *P* = 0.004), independent of LV end-diastolic volume at baseline (*P* = 0.006), whereas CFR was not (*P* = 0.726).

Both IMR and CFR were associated with NT-proBNP at 6 months after adjustment for LV ejection fraction and LV end-diastolic volume at baseline (coefficient [95% CI], 3.39 [0.91, 5.86]; *P* = 0.008 and –111.79 [–195.03, –28.55]; *P* = 0.009, respectively).

### Multivariable associations for IMR and CFR after reperfusion with LV remodeling at 6 months.

IMR was a multivariable associate of changes in LV end-diastolic volume from baseline after adjustment for initial LV end-diastolic volume and clinical characteristics (regression coefficient [95% CI] 0.13 [0.01, 0.24]; *P* = 0.036; Supplemental Table 9), whereas CFR was not (*P* = 0.160).

### Multivariable associations for IMR and CFR after reperfusion with LV ejection fraction at 6 months.

IMR and CFR were multivariable associates of the changes in LV ejection fraction from baseline (Supplemental Table 10).

### Multivariable associations for IMR and CFR adverse health outcomes in the longer term.

All patients (*n* = 283) had long-term follow-up data completed. The median duration of follow-up was of 845 days (after discharge censor duration range, 598–1,098 days). Thirty (11%) patients died or experienced a first heart failure event during the index hospitalization or after discharge. These events included 5 cardiovascular deaths, 3 noncardiovascular deaths, and 22 episodes of heart failure (Killip class 3 or 4 heart failure [*n* = 20] or cardiac device implantation [*n* = 2 received a defibrillator]). Thirteen (4.5%) patients died or experienced a first heart failure hospitalization after discharge, and 8 (61.5%) of these patients had an elevated IMR at baseline.

IMR (per 5-unit difference) was a univariable (hazard ratio [95% CI], 1.08 [1.04, 1.12]; *P* < 0.001) and multivariable (hazard ratio [95% CI], 1.09 [1.04, 1.144]; *P* < 0.001) associate of all-cause death or heart failure hospitalization (Table 6). For a 10-unit difference in IMR, the multivariable hazard ratio for this outcome was 1.18 ([95% CI] 1.09, 1.26) (*P* < 0.001). An IMR >40 was associated with all-cause death or heart failure (4.36 [95% CI], 2.09, 9.06; *P* < 0.001). CFR was not a univariable associate of all-cause death or heart failure hospitalization (CFR, *P* = 0.124).

Forty (14%) patients died or experienced a MACE during the index hospitalization or after discharge. IMR (per 5-unit difference) was a univariable (hazard ratio [95% CI], 1.08 [1.04, 1.11]; *P* < 0.001) and multivariable (hazard ratio [95% CI], 1.07 [1.03, 1.11]; *P* < 0.001) associate of MACE, whereas CFR was not (Table 6).

## Discussion

The main findings of our study are that (a) IMR and CFR can be routinely measured in the culprit coronary artery at the end of emergency PCI; (b) IMR and CFR were both associated with microvascular obstruction and myocardial hemorrhage; (c) compared with IMR, CFR was discriminative of microvascular obstruction in patients with less severe myocardial injury, as reflected by the absence of myocardial hemorrhage, whereas IMR was not discriminative in this group (Figure 3); (d) compared with CFR, IMR was more closely associated with severe vascular damage, as reflected by myocardial hemorrhage, persistent ST-segment elevation, and Killip heart failure classification (Tables 5 and 7); (e) IMR and CFR were associated with proinflammatory cytokines, as revealed by associations with log IL-6 and NT-proBNP; (f) IMR was associated with changes in LV volume at 6 months, whereas CFR was not; (g) IMR was a multivariable associate of adverse health outcome events during longer-term follow-up, whereas CFR was not; and (h) compared with persistent ST-segment elevation >50% on the ECG after reperfusion, an increased IMR had similar predictive value for infarct pathologies (IMR >27) and health outcomes (IMR >40), unlike the duration of symptoms, CFR, and the angiogram.

In line with our hypotheses, IMR and CFR measured in the culprit artery at the end of PCI were multivariable associates of microvascular pathology; however, differences between IMR and CFR were also observed. CFR was discriminative of microvascular function in patients with less severe vascular injury (Figure 3), whereas IMR was not, implying that CFR is more closely associated with reversible microvascular injury. Unlike IMR, CFR was not associated with LV remodeling. These results potentially explain the lack of association between CFR and adverse health outcomes.

The threshold for IMR and outcome has varied between studies (22–24, 28, 29). In our analysis the IMR cut-offs for infarct pathologies and adverse health outcomes differed slightly. An IMR of 27 was most closely associated with microvascular obstruction and myocardial hemorrhage, whereas a higher IMR of 40 was most closely associated with all-cause death or heart failure. We think that the higher IMR cut-off for the occurrence of adverse health outcomes reflects the pathophysiological consequences of infarct pathologies, and so the higher cut-off for clinical outcomes intuitively makes sense. Our paper adds to the literature on IMR in patients with acute STEMI (22–24, 28, 29) and provides pathophysiologic and clinical data in support of the validity of IMR as a direct invasive test of myocardial reperfusion (30).

The 12-lead surface ECG 60 to 90 minutes after reperfusion is the standard of care diagnostic test for assessing the efficacy of coronary reperfusion (1). Our results provide further pathophysiologic validation of the ECG. However, ECG evidence of failed myocardial reperfusion occurred in a minority of patients (14% for <30% ST-segment resolution; 28% for <50% ST-segment resolution). Consequently, the surface ECG resulted in a missed diagnosis of failed myocardial reperfusion in almost half of all comers in whom failed microvascular reperfusion had occurred, as revealed by cardiac MRI (reference test). In multivariate analysis of the predictors of all-cause death or heart failure, both IMR (for a 5-unit change) and <50%ST- segment resolution were predictors of this outcome (Table 6). The diagnostic and prognostic value of the ECG varied markedly between <30% ST-segment resolution and <50% ST-segment resolution. The specificity (rule-in test) and negative predictive values (rule-out test) for the different ECG cut-offs differed markedly, whereas the predictive values of IMR for cut-offs 27 and 40 for all of the outcomes were broadly similar. Usually, patients have returned to the ward by the time of the 90-minute ECG, limiting the options for more intensive therapy. On the other hand, IMR is a direct, invasive test that can be used to assess the efficacy of myocardial reperfusion.

TIMI coronary flow grades before but not after PCI were associated with IMR (Table 1) and microvascular pathology (Table 3). Microvascular obstruction occurred in half of the study participants, despite successful restoration of epicardial coronary artery flow in the majority (99% in our population, in line with previous reports; refs. 7–9). Furthermore, TIMI blush grades were not associated with microvascular pathology or adverse health outcomes in the longer term (Tables 4–7). TIMI blush grades require high-quality angiography and expertise for reliable assessment. Blush grades have applications for research purposes rather than in routine practice (1, 3).

Overall, IMR was more closely associated with severe irreversible infarct pathology, i.e., myocardial hemorrhage and subsequent adverse LV and clinical outcomes, than other tests of myocardial reperfusion. In clinical practice involving patients with acute STEMI, microvascular obstruction and myocardial hemorrhage routinely pass undetected, despite having proven prognostic value. We think that IMR has potential to address this disparity in diagnosis. The high negative predictive value of an IMR >27 for myocardial hemorrhage points to its potential value as an early rule-out test for failed myocardial reperfusion. On the other hand, we do not propose IMR as an alternative for CMR, which is performed subsequently downstream in the care pathway as a test for LV function, pathology, and viability (1, 3).

In STEMI patients with less severe vascular injury, as revealed by the absence of myocardial hemorrhage, CFR was associated with microvascular obstruction, which is a potentially reversible pathology (6, 31). This observation is consistent with the notion that CFR reflects coronary artery function and vasoreactivity. However, CFR was not associated with LV remodeling or adverse health outcomes in the longer term, limiting its clinical utility. By contrast, IMR, which is a quantitative parameter of microvascular resistance, was associated with IL-6 concentrations, notably in the subgroup of patients with myocardial hemorrhage. The association between IMR and IL-6 provides a mechanistic explanation. In the setting of acute reperfused STEMI, an elevated IMR measured in the culprit coronary artery after reperfusion reflects severe microvascular dysfunction. The association between IMR and subsequent systemic concentrations of IL-6 on the first day after STEMI reflects systemic inflammation and vascular injury, reflected by the occurrence of myocardial hemorrhage within the infarct core. These pathophysiologic insights potentially explain the strong associations between IMR measured acutely and surrogate outcomes, including adverse LV remodeling, and all-cause death and heart failure early after STEMI.

Our study adds to previous investigations of IMR and CFR in patients with acute STEMI. Van de Hoef et al. (32) found that, in 148 STEMI survivors, a CFR <2.0 measured in the culprit coronary artery was associated with MACE over 10 years after STEMI. The prognostic significance of IMR in patients with acute STEMI is more established. IMR is independently associated with LV function (33), infarct pathology (24, 28), and health outcomes after STEMI (23). In a pooled analysis of 253 patients with acute STEMI followed for a median of 2.8 years, Fearon et al. (23) found that an IMR >40 was a multivariable associate of all-cause death and heart failure, whereas CFR was not. Other comparative studies of CFR and IMR are limited by sample size (*n* = 27–45) (22, 29, 33, 34) and follow-up duration (3–6 months) (22, 29, 33, 34). Taken together, our study adds importantly to what is already known. We describe the largest study to date involving invasive measurements of microvascular resistance in the culprit artery, infarct characterization using cardiac MRI 2 days and 6 months later, and longitudinal follow-up for surrogate outcomes and all-cause death or heart failure events during longer-term follow-up.

Cuculi et al. recently reported that CFR and IMR are modifiable (22). They found that the change in CFR within 24 hours of reperfusion is associated with infarct size and myocardial salvage, which is consistent with previous observations and those in our study (24, 28, 33). Further studies are warranted to determine whether IMR (with or without CFR) might be a clinically useful biomarker to risk-stratify patients for targeted therapy, since practice guidelines identify failed myocardial reperfusion as a clinical problem of unmet need (1).

The study population included 21 patients initially treated with thrombolysis, and 14 of these patients had rescue PCI. The main results of our study were unchanged when these patients were removed (data not shown), and myocardial hemorrhage was similar in patients treated by primary PCI versus thrombolysis. We conclude that guide wire–based assessment of coronary microvascular function is of diagnostic value in patients treated with thrombolysis.

### Limitations.

We performed a single-center natural history study involving prospective enrollment of patients with acute STEMI. We did not enroll consecutive patients (Figure 2). Coronary venous blood samples were not collected based on safety and logistics. Invasive assessments of culprit artery microvascular function and contrast-enhanced MRI were obtained in 283 patients (*n* = 281 with both CFR and IMR); however, evaluable T2*maps were not evaluable in all of these subjects due to cardiorespiratory motion and breath-holding problems in acutely ill patients. Enrollment in our study took place 24/7; however, blood sample handling for NT-proBNP and IL-6 was not feasible outside of collection hours. The clinical characteristics of the patients in whom blood samples were obtained were similar to the whole population (Supplemental Results).

The limited number of adverse events constrained the number of variables and statistical power in the Cox models. Our study builds on previous investigations in smaller patient cohorts. Although our study does not include a validation cohort, the prognostic importance of IMR is consistent with previous studies involving IMR in STEMI patients (23). Our analysis does not permit inference on causality, and further studies are warranted.

### Conclusions.

Our results support the feasibility and pathophysiological validity of IMR, over CFR and the ECG, as a routine diagnostic test for failed myocardial reperfusion in patients with acute STEMI. Routine measurement of IMR has potential clinical utility for immediate stratification of patients with acute STEMI following coronary reperfusion, and further research is warranted.

## Methods

### Participants and interventions

We performed a prospective cohort study in a single regional cardiac center between July 14, 2011, and November 22, 2012. Two hundred and eighty-eight STEMI patients provided written informed consent to undergo a diagnostic guide wire–based assessment after reperfusion and then MRI 2 days and 6 months later as well as follow-up for health outcomes in the longer term. Blood samples were obtained in a subset of patients during office hours (day 1 and 6 months after STEMI) for measurement of IL-6 and NT-proBNP, a biochemical measure of LV wall stress. Patients were eligible if they had an indication for primary PCI or thrombolysis for acute STEMI due to a history of symptoms consistent with acute myocardial ischemia and with supporting changes on the ECG (i.e., ST-segment elevation or new left bundle-branch block) (1). Exclusion criteria represented standard contraindications to contrast MRI, including a pacemaker and estimated glomerular filtration rate <30 ml/min/1.73 m^2^. Acute STEMI management followed contemporary guidelines (1, 3). Aspiration thrombectomy, direct stenting, antithrombotic drugs, and other therapies were administered according to clinical judgment (Supplemental Methods). The ClinicalTrials.gov identifier for this study is NCT02072850, and the study design conforms with CONSORT, STROBE, and TREND guidelines.

### Measurement of IMR and CFR at the end of PCI

A pressure- and temperature-sensitive coronary guide wire (St. Jude Medical, Minnesota, USA) was used to measure IMR and CFR in the culprit coronary artery at the end of primary or rescue PCI. The guide wire was calibrated outside the body, equalized with aortic pressure at the ostium of the guide catheter, and then advanced to the distal third of the culprit artery. CFR is defined as the mean transit time at rest divided by the mean transit time during hyperemia. IMR is defined as the distal coronary pressure multiplied by the mean transit time of 3 sequential manual bolus injections of saline (3 ml) at room temperature during maximal coronary hyperemia, measured simultaneously (mmHg × s or units) (24, 28, 33).

Hyperemia was induced by 140 μ/kg/min of i.v. adenosine preceded by a 2-ml intracoronary bolus of 200 μg of nitrate. The mean aortic and distal coronary pressures were recorded during maximal hyperemia. In our study, the repeatability of CFR and IMR was assessed by duplicate measurements 5 minutes apart in a subset of 12 consecutive patients, in line with previous observations (28). We have previously observed that repeated IMR measurements obtained by 4 different operators in 12 STEMI patients were highly correlated (r = 0.99, *P* < 0.001), with a mean difference between IMR measurements of 0.01 (mean standard error 1.59 [95% CI −3.52 to 3.54], *P* = 0.48) (28).

### Cardiac MRI acquisition

MRI was performed on a Siemens MAGNETOM Avanto 1.5-Tesla scanner with a 12-element phased array cardiac surface coil (35). The imaging protocol (5, 36) included cine MRI with steady-state free precession (SSFP), T2 mapping (37, 38), T2*-mapping, and delayed-enhancement phase-sensitive inversion-recovery pulse sequences (39). The scan acquisitions were spatially coregistered and also included different slice orientations to enhance diagnostic confidence.

T2 maps were acquired in contiguous short-axis slices covering the whole ventricle, using an investigational prototype T2-prepared TrueFisp sequence (37, 38) (Supplemental Methods). The hypointense infarct core on T2 mapping reflects the combination of microvascular obstruction and myocardial hemorrhage (4). Typical imaging parameters were as follows: bandwidth, approximately 947 Hz/pixel; flip angle, 70°; T2 preparations, 0 ms, 24 ms, and 55 ms, respectively; matrix, 160 × 105 pixels; spatial resolution, 2.6 × 2.1 × 8.0 mm; and slice thickness, 8 mm.

Myocardial hemorrhage was detected using an investigational prototype T2* map sequence acquired in 3 short-axis slices (basal, mid, and apical). Typical imaging parameters were as follows: bandwidth, approximately 814 (×8) Hz/pixel; flip angle, 18°; matrix, 256 × 115; spatial resolution, 2.6 × 1.6 × 10 mm; and slice thickness, 8 mm. This method became available for use after 25 patients had been enrolled.

Early gadolinium enhancement (EGE) imaging was acquired 1, 3, 5, and 7 minutes after contrast injection using a TrueFISP readout and a fixed inversion time of 440 ms. Late gadolinium enhancement images covering the entire LV were acquired 10 to 15 minutes after i.v. injection of 0.15 mmol/kg of gadoterate meglumine (Gd^2+^-DOTA, Dotarem, Guerbet S.A.) using segmented phase-sensitive inversion recovery turbo fast low-angle shot sequence (39). Typical imaging parameters were as follows: matrix, 192 × 256; flip angle, 25°; TE, 3.36 ms; bandwidth, 130 Hz/pixel; echo spacing, 8.7 ms; and trigger pulse, 2. The voxel size was 1.8 × 1.3 × 8 mm^3^. Inversion times were individually adjusted to optimize nulling of apparently normal myocardium (typical values, 200 to 300 ms).

### Cardiac MRI analyses

The images were analyzed on a Siemens work station by observers with at least 3 years MRI experience (N. Ahmed, D. Carrick, I. Mordi, S.M.O. Rauhalammi). All of the images were reviewed by an experienced MRI cardiologist (C. Berry). LV dimensions, volumes, and ejection fractions were quantified using computer-assisted planimetry (syngo MR, Siemens Healthcare). The late gadolinium enhancement images were analyzed for infarct size and microvascular obstruction by observers (N. Ahmed, I. Mordi) who were blinded to all of the other data. In healthy volunteers, the absence of late gadolinium enhancement was determined qualitatively by visual assessment.

In STEMI patients, myocardial T2/T2* values were segmented spatially and regions of interest were defined as (a) remote myocardium, (b) injured myocardium, and (c) infarct core. The regions of interest were planimetered to include the entire area of interest, with distinct margins of separation from tissue interfaces to avoid partial volume averaging. The remote myocardium region of interest was defined as myocardium 180° from the affected zone with no visible evidence of infarction, edema, or wall motion abnormalities (assessed by inspecting corresponding contrast-enhanced T1-weighted, T2-weighted, and cine images, respectively). The infarct zone region of interest was defined as myocardium with pixel values (T2) >2 SD from remote myocardium on T2-weighted MRI (37, 38). The hypointense infarct core was defined as an area in the center of the infarct territory having a mean T2* value of at least 2 SDs below the T2* value of the periphery of the area at risk (37, 38). The assessment of T2* maps and adjudication (present/absent) of a hypointense core was performed independently by D. Carrick.

#### Infarct definition and size.

The presence of acute infarction was established based on abnormalities in cine wall motion, rest first-pass myocardial perfusion, and delayed-enhancement imaging in two imaging planes. In addition, supporting changes on the ECG and coronary angiogram were also required. Acute infarction was considered present only if late gadolinium enhancement was confirmed on both the axial and long-axis acquisitions. The myocardial mass of late gadolinium (grams) was quantified using computer-assisted planimetry, and the territory of infarction was delineated using a signal intensity threshold of >5 SDs above a remote reference region and expressed as a percentage of total LV mass (40). Infarct regions with evidence of microvascular obstruction were included within the infarct area, and the extent of microvascular LV ventricular mass was also measured. The measurements of infarct size were performed by I. Mordi and N. Ahmed.

#### Microvascular obstruction.

Microvascular obstruction was defined as a dark zone on EGE imaging 1, 3, 5, and 7 minutes after contrast injection that remained present within an area of large gadolinium enhancement at 15 minutes. Identification of microvascular obstruction was performed independently by I. Mordi and N. Ahmed.

#### Extent of myocardial edema.

The extent of myocardial edema representing the jeopardized area at risk was defined as LV myocardium with pixel values (T2) >2 SDs from remote myocardium (37, 38, 41–44). In order to assess the area at risk, the epicardial and endocardial contours on the last corresponding T2-weighted raw image with an echo time of 55 ms were planimetered (37). Contours were then copied to the computed T2 map and corrected when necessary by consulting the SSFP cine images.

#### Myocardial hemorrhage.

On the T2* maps, a region of reduced signal intensity within the infarcted area with a T2* value of <20 ms (45–48) was considered to confirm the presence of myocardial hemorrhage.

### ECG

A 12-lead ECG was obtained before coronary reperfusion and 60 minutes afterwards. The extent of ST-segment resolution on the ECG assessed 60 minutes after reperfusion compared to the baseline ECG before reperfusion (49) was expressed as successful ≥50% ST-segment resolution on the ECG 60 minutes after reperfusion or or a lack of <50% ST-segment resolution. Additionally the ST-segment resolution was categorized as follows: complete (~70%), incomplete (30% to <70%), or none (<30%).

### Biochemical and hematology measurements

Serial systemic blood samples were obtained immediately after reperfusion in the cardiac catheterization laboratory and subsequently on the first day (0600–0700 hours) during the initial in-patient stay in the Coronary Care Unit.

CRP was measured in an NHS hospital biochemistry laboratory using a particle-enhanced immunoturbimetric assay method (Cobas C501, Roche) and the manufacturer’s calibrators and quality control material, as a biochemical measure of inflammation. The high-sensitivity CRP assay measurement range was 0.1–250 mg/l. The expected CRP values in a healthy adult are <5 mg/l, and the reference range in our hospital is 0–10 mg/l. IL-6 was measured using a high-sensitivity ELISA (R&D Systems) (50). The limit of detection was <0.1 pg/ml, and the intraassay CV was 9.1%. NT-proBNP was measured in a research laboratory using an electrochemiluminescence method (e411, Roche), and the manufacturer’s calibrators and quality control material. The limit of detection for IL-6 and NT-proBNP is 6.5 pg/ml and 5 pg/ml, respectively. Long-term coefficient of variations of low and high controls are typically <5% and were all within the manufacturer’s range.

### Prespecified health outcomes

We prespecified adverse health outcomes that are pathophysiologically linked with STEMI. The primary composite outcome was (1) all-cause death or first heart failure event following the initial hospitalization (Supplemental Methods).

Research staff screened for events from enrollment by checking the medical records and by contacting patients and their primary and secondary care physicians as appropriate, with no loss to follow-up (Figure 2). Each serious adverse event was reviewed by a cardiologist who was independent of the research team and blinded to all of the clinical and MRI data. The serious adverse events were defined according to standard guidelines (refs. 51, 52, and Supplemental Methods) and categorized as having occurred either during the index admission or after discharge. All study participants were followed up for a minimum of 18 months after discharge. The median duration of follow-up was 845 days (after discharge censor duration range, 598–1,098 days).

### Blinding

The study participants were blinded to all of the clinical assessments, including CFR, IMR, ECG, and MRI results. The observers who analyzed the surrogate outcomes and those who adjudicated the adverse clinical events were blinded to all of the other clinical data.

### Statistics

The sample size calculation is described in the Supplemental Methods. We estimated that at least 30 all-cause death or heart failure events would occur based on a conservative estimate of the event rate (10%–12%) at 18 months.

Categorical variables are expressed as number and percentage of patients. Most continuous variables followed a normal distribution and are therefore presented as means together with SD. Those variables that did not follow a normal distribution are presented as medians with IQR. Differences in continuous variables between groups were assessed by 2-tailed Student’s *t* test or ANOVA for continuous data with normal distribution; otherwise, the nonparametric Wilcoxon rank-sum test or Kruskal-Wallis test was used. Differences in categorical variables between groups were assessed using a χ^2^ test or Fisher’s test, as appropriate. Correlation analyses were performed using Pearson or Spearman tests, as indicated. Random effects models were used to compute interrater and intrarater reliability measures (intraclass correlation coefficient [ICC]) for the reliability of CFR and IMR values measured independently by 2 observers in 12 randomly selected patients from the cohort. In addition, ICC was measured for the reliability of infarct core T2* values measured independently by 2 observers in 20 randomly selected patients from the cohort.

Univariable and multivariable linear regression methods to identify associates of CFR and IMR values for infarct pathology revealed by MRI (infarct size, EGE, late microvascular obstruction, and myocardial hemorrhage) and circulating cytokines (IL-6, NT-proBNP) are described in the Supplemental Methods.

Receiver operating curve, Kaplan-Meier, and Cox proportional hazards methods were used to identify potential clinical predictors of all-cause death/heart failure events, including patient characteristics, MRI findings, and IMR/CFR.

All *P* values were 2 sided, and *P* > 0.05 indicates the absence of a statistically significant effect. Statistical analyses were performed using R v 2.15.1 or SAS v 9.3 or higher versions of these programs.

### Study approval

The study was approved by the UK National Research Ethics Service (reference 10-S0703-28). All of the participants provided witnessed informed assent at the time of the acute procedure followed by written informed consent on the ward.

## Author contributions

DC coordinated the study, obtained informed consent from all of the participants, and coordinated and analyzed the MRI scans. He collected the clinical data, participated in the statistical analyses, interpreted the results, and drafted the manuscript. MM, MCP, HE, ML, SW, and SH obtained informed assent, collected data, interpreted the results, and contributed to the manuscript. CH and IF contributed to study design, analyzed and interpreted the data, and contributed to the manuscript. AM assessed the source data for serious adverse events during follow-up that were potentially relevant to the prespecified health outcomes. IM, NA, SMOR, and AR contributed to the analysis of the MRI scans in STEMI patients and in healthy volunteers and contributed to the manuscript. PM analyzed the ECGs. JC and VTYM undertook quantitative coronary analysis of the angiograms. PW and NS undertook the analysis of blood samples for NT-proBNP and IL-6 and contributed to the manuscript. KGO helped to conceive the idea for the study, collected data, interpreted the results, and contributed to the manuscript. CB is PI for the British Heart Foundation project grant and chief investigator for the clinical study. CB conceived the idea for the study and obtained the funding and IRB approvals. He participated in patient recruitment, collected clinical data, interpreted the results, and jointly wrote the manuscript. CB takes responsibility for the manuscript.

## Supplementary Material

Supplemental data

ICMJE disclosure forms

## Figures and Tables

**Table 7:**
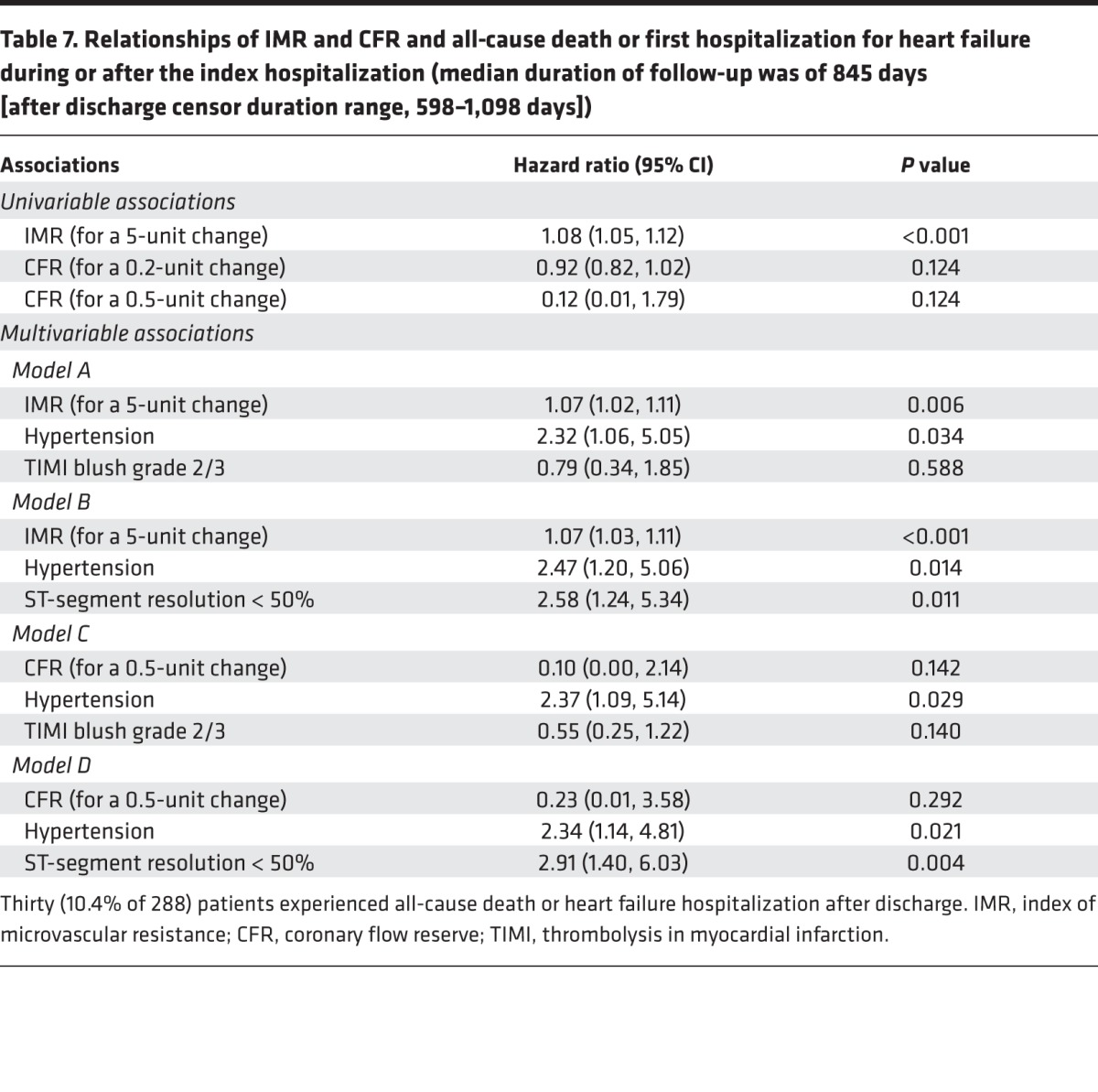
Relationships of IMR and CFR and all-cause death or first hospitalization for heart failure during or after the index hospitalization (median duration of follow-up was of 845 days [after discharge censor duration range, 598–1,098 days])

**Table 6:**
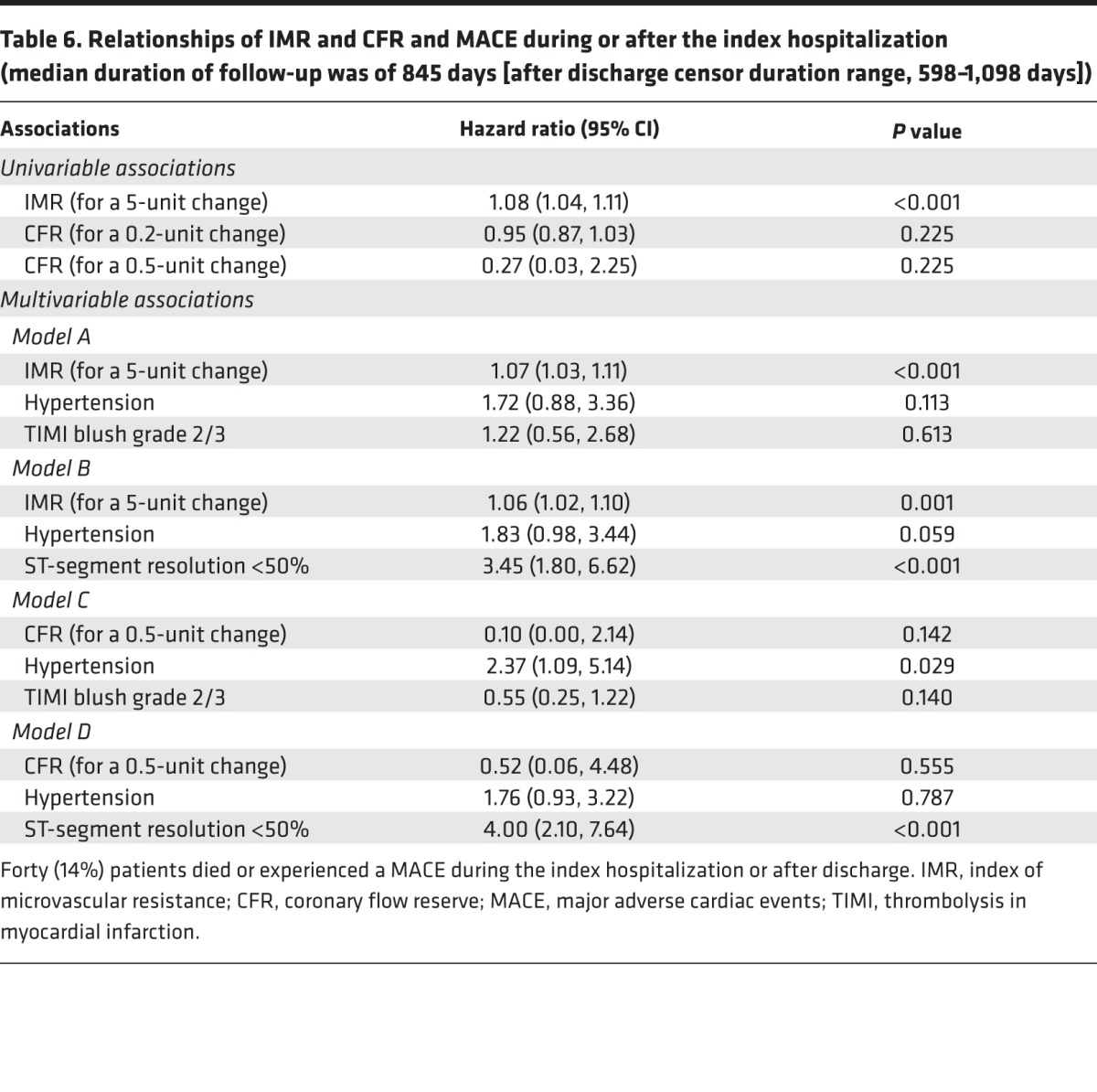
Relationships of IMR and CFR and MACE during or after the index hospitalization (median duration of follow-up was of 845 days [after discharge censor duration range, 598–1,098 days])

**Table 5:**
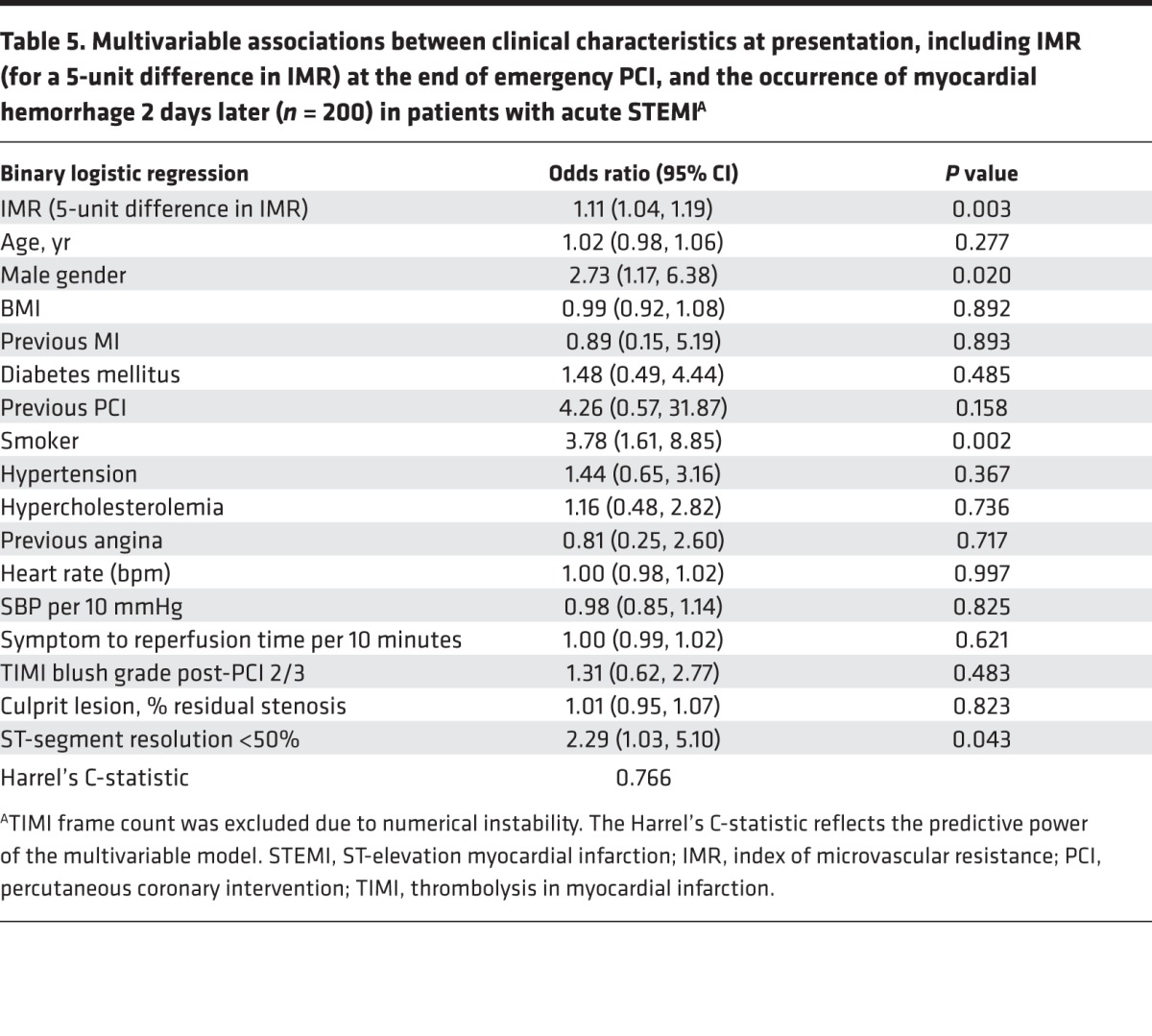
Multivariable associations between clinical characteristics at presentation, including IMR (for a 5-unit difference in IMR) at the end of emergency PCI, and the occurrence of myocardial hemorrhage 2 days later (*n* = 200) in patients with acute STEMI^A^

**Table 4:**
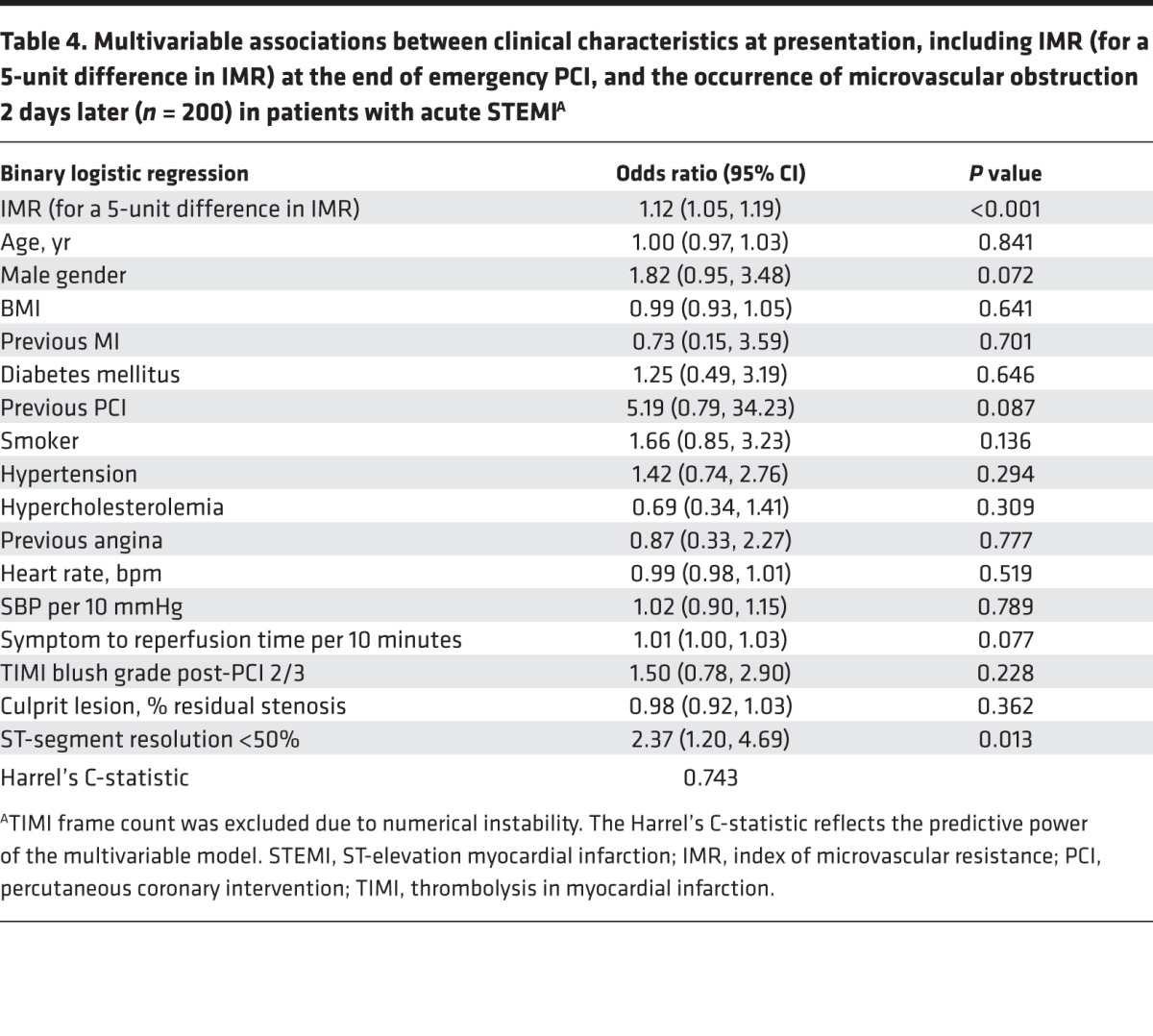
Multivariable associations between clinical characteristics at presentation, including IMR (for a 5-unit difference in IMR) at the end of emergency PCI, and the occurrence of microvascular obstruction 2 days later (*n* = 200) in patients with acute STEMI^A^

**Table 3:**
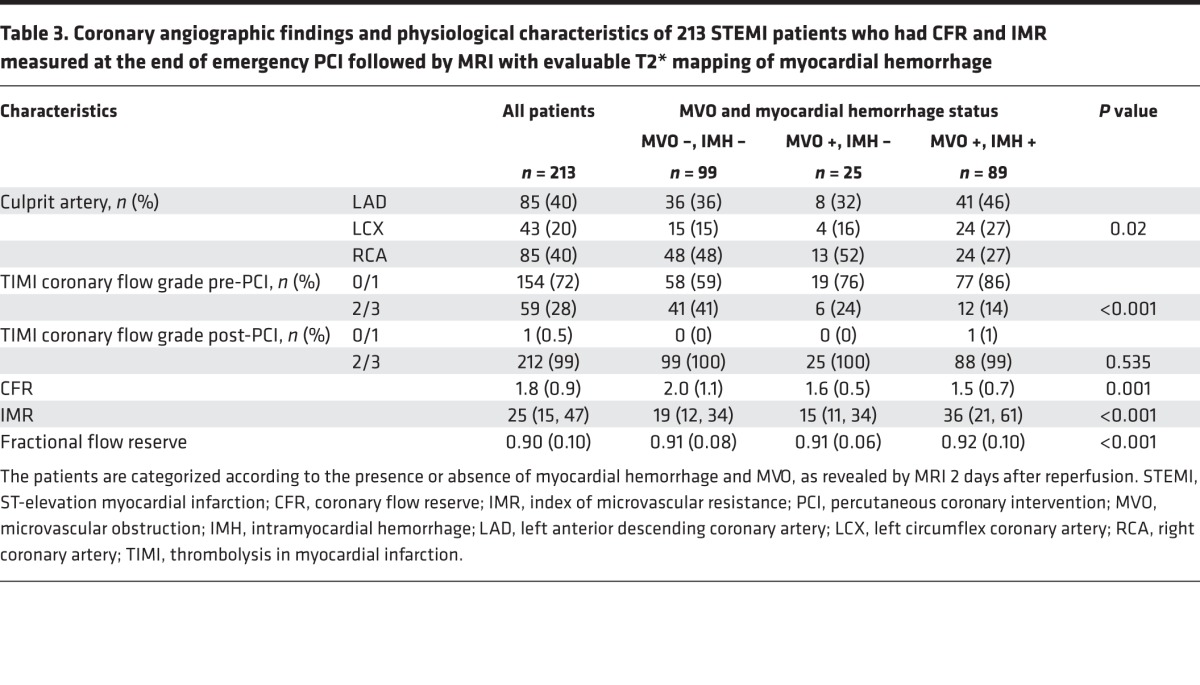
Coronary angiographic findings and physiological characteristics of 213 STEMI patients who had CFR and IMR measured at the end of emergency PCI followed by MRI with evaluable T2* mapping of myocardial hemorrhage

**Table 2:**
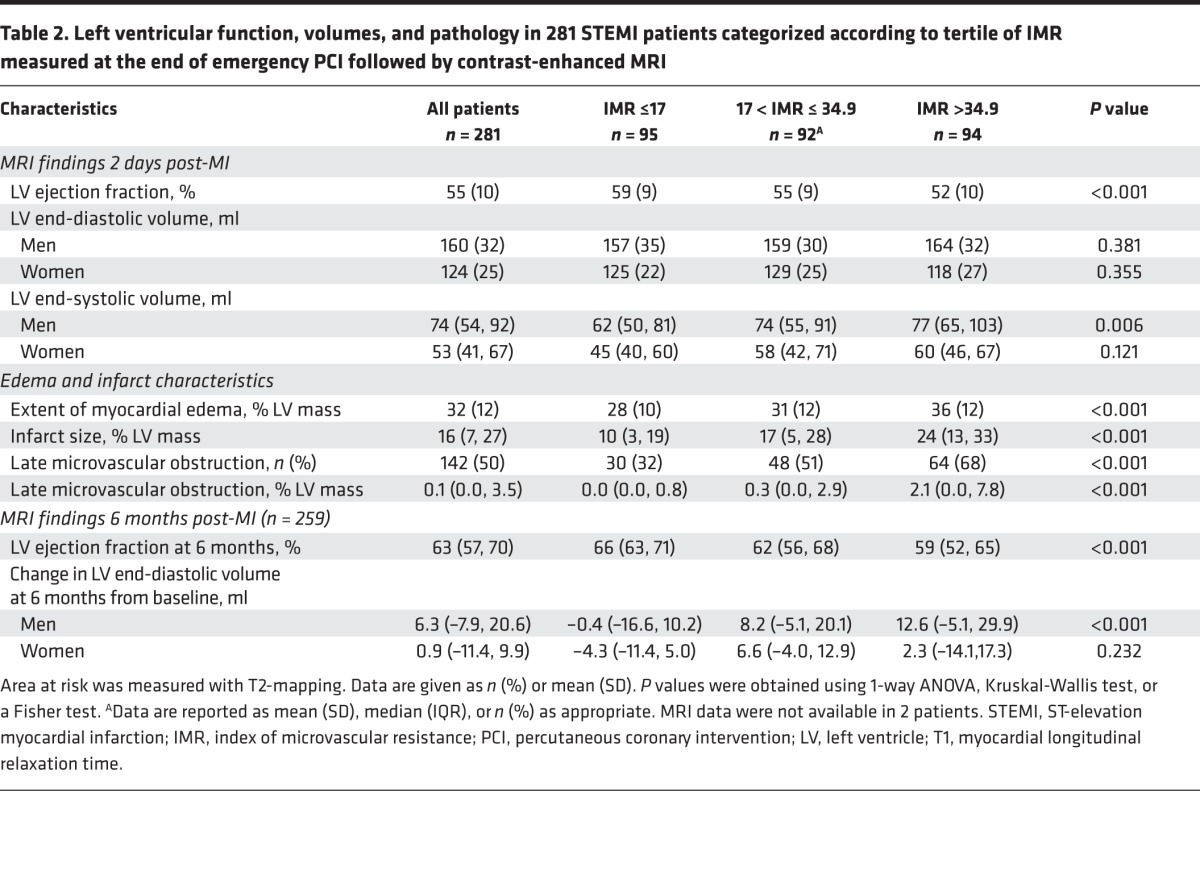
Left ventricular function, volumes, and pathology in 281 STEMI patients categorized according to tertile of IMR measured at the end of emergency PCI followed by contrast-enhanced MRI

**Table 1:**
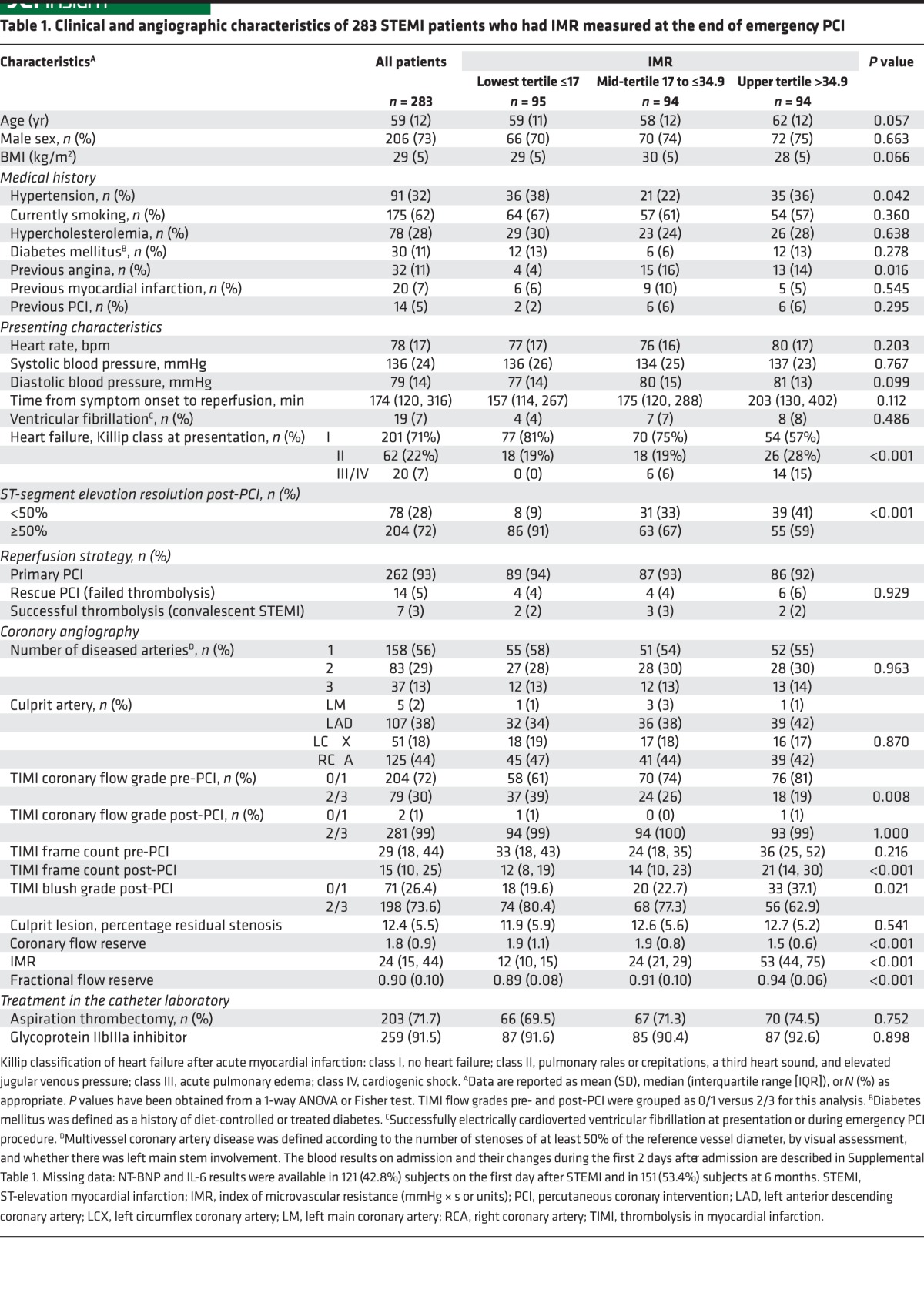
Clinical and angiographic characteristics of 283 STEMI patients who had IMR measured at the end of emergency PCI

## References

[1] O’Gara PT (2013). 2013 ACCF/AHA guideline for the management of ST-elevation myocardial infarction: a report of the American College of Cardiology Foundation/American Heart Association Task Force on Practice Guidelines. Circulation.

[2] Mozaffarian D (2015). Heart disease and stroke statistics — 2015 update: a report from the American Heart Association. Circulation.

[3] Levine GN (2011). 2011 ACCF/AHA/SCAI Guideline for Percutaneous Coronary Intervention. A report of the American College of Cardiology Foundation/American Heart Association Task Force on Practice Guidelines and the Society for Cardiovascular Angiography and Interventions. J Am Coll Cardiol.

[4] British Cardiovascular Intervention Society Audit for Adult Cardiovascular Interventions. BCIS Web site.

[5] Carrick D (2016). Prognostic significance of infarct core pathology revealed by quantitative non-contrast in comparison with contrast cardiac magnetic resonance imaging in reperfused ST-elevation myocardial infarction survivors. Eur Heart J.

[6] Robbers LF (2013). Magnetic resonance imaging-defined areas of microvascular obstruction after acute myocardial infarction represent microvascular destruction and haemorrhage. Eur Heart J.

[7] Ahmed N, Carrick D, Layland J, Oldroyd KG, Berry C (2013). The role of cardiac magnetic resonance imaging (MRI) in acute myocardial infarction (AMI). Heart Lung Circ.

[8] Carrick D, Berry C (2013). Prognostic importance of myocardial infarct characteristics. Eur Heart J Cardiovasc Imaging.

[9] Frohlich GM, Meier P, White SK, Yellon DM, Hausenloy DJ (2013). Myocardial reperfusion injury: looking beyond primary PCI. Eur Heart J.

[10] Chen J, Hsieh AF, Dharmarajan K, Masoudi FA, Krumholz HM (2013). National trends in heart failure hospitalization after acute myocardial infarction for Medicare beneficiaries: 1998–2010. Circulation.

[11] Ganame J (2009). Impact of myocardial haemorrhage on left ventricular function and remodelling in patients with reperfused acute myocardial infarction. Eur Heart J.

[12] Eitel I (2011). Prognostic value and determinants of a hypointense infarct core in T2-weighted cardiac magnetic resonance in acute reperfused ST-elevation-myocardial infarction. Circ Cardiovasc Imaging.

[13] van Kranenburg M (2014). Prognostic value of microvascular obstruction and infarct size, as measured by CMR in STEMI patients. JACC Cardiovasc Imaging.

[14] de Waha S (2010). Impact of early vs. late microvascular obstruction assessed by magnetic resonance imaging on long-term outcome after ST-elevation myocardial infarction: a comparison with traditional prognostic markers. Eur Heart J.

[15] van ‘t Hof AW, Liem A, Suryapranata H, Hoorntje JC, de Boer MJ, Zijlstra F (1998). Angiographic assessment of myocardial reperfusion in patients treated with primary angioplasty for acute myocardial infarction: myocardial blush grade. Zwolle Myocardial Infarction Study Group. Circulation.

[16] Gibson CM (2000). Relationship of TIMI myocardial perfusion grade to mortality after administration of thrombolytic drugs. Circulation.

[17] Barbato E (2004). Validation of coronary flow reserve measurements by thermodilution in clinical practice. Eur Heart J.

[18] Gould KL, Hamilton GW, Lipscomb K, Ritchie JL, Kennedy JW (1974). Method for assessing stress-induced regional malperfusion during coronary arteriography. Experimental validation and clinical application. Am J Cardiol.

[19] Fearon WF (2004). Microvascular resistance is not influenced by epicardial coronary artery stenosis severity: experimental validation. Circulation.

[20] Ng MK, Yeung AC, Fearon WF (2006). Invasive assessment of the coronary microcirculation: superior reproducibility and less hemodynamic dependence of index of microcirculatory resistance compared with coronary flow reserve. Circulation.

[21] Johnson NP, Kirkeeide RL, Gould KL (2013). Coronary anatomy to predict physiology: fundamental limits. Circ Cardiovasc Imaging.

[22] Cuculi F (2014). Impact of microvascular obstruction on the assessment of coronary flow reserve, index of microcirculatory resistance, and fractional flow reserve after ST-segment elevation myocardial infarction. J Am Coll Cardiol.

[23] Fearon WF (2013). Prognostic value of the Index of Microcirculatory Resistance measured after primary percutaneous coronary intervention. Circulation.

[24] McGeoch R (2010). The index of microcirculatory resistance measured acutely predicts the extent and severity of myocardial infarction in patients with ST-segment elevation myocardial infarction. JACC Cardiovasc Interv.

[25] Neumann FJ (1995). Cardiac release of cytokines and inflammatory responses in acute myocardial infarction. Circulation.

[26] Guillen I, Blanes M, Gomez-Lechon MJ, Castell JV (1995). Cytokine signaling during myocardial infarction: sequential appearance of IL-1 beta and IL-6. Am J Physiol.

[27] Echavarria-Pinto M (2013). Disturbed coronary hemodynamics in vessels with intermediate stenoses evaluated with fractional flow reserve: a combined analysis of epicardial and microcirculatory involvement in ischemic heart disease. Circulation.

[28] Payne AR (2012). Microvascular resistance predicts myocardial salvage and infarct characteristics in ST-elevation myocardial infarction. J Am Heart Assoc.

[29] Sezer M (2010). Concurrent microvascular and infarct remodeling after successful reperfusion of ST-elevation acute myocardial infarction. Circ Cardiovasc Interv.

[30] Ahmed N (2015). Safety of guidewire-based measurement of fractional flow reserve and the index of microvascular resistance using intravenous adenosine in patients with acute or recent myocardial infarction. Int J Cardiol.

[31] Carrick D (2016). Myocardial hemorrhage after acute reperfused ST-segment-elevation myocardial infarction: relation to microvascular obstruction and prognostic significance. Circ Cardiovasc Imaging.

[32] van de Hoef TP (2014). Physiological basis and long-term clinical outcome of discordance between fractional flow reserve and coronary flow velocity reserve in coronary stenoses of intermediate severity. Circ Cardiovasc Interv.

[33] Fearon WF (2008). Predictive value of the index of microcirculatory resistance in patients with ST-segment elevation myocardial infarction. J Am Coll Cardiol.

[34] Kitabata H (2009). Coronary microvascular resistance index immediately after primary percutaneous coronary intervention as a predictor of the transmural extent of infarction in patients with ST-segment elevation anterior acute myocardial infarction. JACC Cardiovasc Imaging.

[35] Kramer CM, Barkhausen J, Flamm SD, Kim RJ, Nagel E (2013). Standardized cardiovascular magnetic resonance (CMR) protocols 2013 update. J Cardiovasc Magn Reson.

[36] Carrick D (2015). Pathophysiology of LV remodeling in survivors of STEMI: inflammation, remote myocardium, and prognosis. JACC Cardiovasc Imaging.

[37] Giri S (2009). T2 quantification for improved detection of myocardial edema. J Cardiovasc Magn Reson.

[38] Verhaert D (2011). Direct T2 quantification of myocardial edema in acute ischemic injury. JACC Cardiovasc Imaging.

[39] Kellman P, Arai AE, McVeigh ER, Aletras AH (2002). Phase-sensitive inversion recovery for detecting myocardial infarction using gadolinium-delayed hyperenhancement. Magn Reson Med.

[40] Flett AS (2011). Evaluation of techniques for the quantification of myocardial scar of differing etiology using cardiac magnetic resonance. JACC Cardiovasc Imaging.

[41] Eitel I (2010). Prognostic significance and determinants of myocardial salvage assessed by cardiovascular magnetic resonance in acute reperfused myocardial infarction. J Am Coll Cardiol.

[42] Berry C (2010). Magnetic resonance imaging delineates the ischemic area at risk and myocardial salvage in patients with acute myocardial infarction. Circ Cardiovasc Imaging.

[43] Payne AR (2011). Bright-blood T2-weighted MRI has higher diagnostic accuracy than dark-blood short tau inversion recovery MRI for detection of acute myocardial infarction and for assessment of the ischemic area at risk and myocardial salvage. Circ Cardiovasc Imaging.

[44] Francone M (2009). Impact of primary coronary angioplasty delay on myocardial salvage, infarct size, and microvascular damage in patients with ST-segment elevation myocardial infarction: insight from cardiovascular magnetic resonance. J Am Coll Cardiol.

[45] Ghugre NR (2011). Quantitative tracking of edema, hemorrhage, and microvascular obstruction in subacute myocardial infarction in a porcine model by MRI. Magn Reson Med.

[46] Kandler D (2014). The relation between hypointense core, microvascular obstruction and intramyocardial haemorrhage in acute reperfused myocardial infarction assessed by cardiac magnetic resonance imaging. Eur Radiol.

[47] O’Regan DP, Ariff B, Neuwirth C, Tan Y, Durighel G, Cook SA (2010). Assessment of severe reperfusion injury with T2* cardiac MRI in patients with acute myocardial infarction. Heart.

[48] Anderson LJ (2001). Cardiovascular T2-star (T2*) magnetic resonance for the early diagnosis of myocardial iron overload. Eur Heart J.

[49] Steg PG (2012). ESC Guidelines for the management of acute myocardial infarction in patients presenting with ST-segment elevation. Eur Heart J.

[50] Murray ET (2015). Overweight across the life course and adipokines, inflammatory and endothelial markers at age 60-64 years: evidence from the 1946 birth cohort. Int J Obes (Lond).

[51] Thygesen K (2012). Third niversal definition of myocardial infarction. Eur Heart J.

[52] Hicks KA (2015). 2014 ACC/AHA Key Data Elements and Definitions for Cardiovascular Endpoint Events in Clinical Trials: A Report of the American College of Cardiology/American Heart Association Task Force on Clinical Data Standards (Writing Committee to Develop Cardiovascular Endpoints Data Standards). J Am Coll Cardiol.

