# Supplementary material for: Microvascular resistance of the culprit coronary artery in acute ST-elevation myocardial infarction
Source: JCI Insight. 2016 May 5;1(6):e85768. doi: 10.1172/jci.insight.85768 (PMC5033815; doi:10.1172/jci.insight.85768)

Supplementary Results

Microvascular function in the culprit coronary artery in acute ST-elevation myocardial infarction.

ClinicalTrials.gov registration NCT02072850

Intra- and inter-observer agreement of T2* core (hemorrhage) measurements

Mean T2* relaxation times (ms) in regions-of-interest in the infarct of 20 randomly chosen patients were independently measured by two observers. The intra-class correlation coefficient for reliability of infarct core T2 is: 0.90 (0.77, 0.96); $p<0.001$. Bland-Altman plot showed no evidence of bias (supplementary figure).

Table 1. Blood results on admission and medical therapy on discharge in 283 STEMI patients who had IMR measured at the end of emergency PCI.

Characteristics*		All patients	Index of microvascular resistance, IMR			P-value
			Lowest tertile ≤ 17	Mid-tertile 17 to ≤ 34.9	Upper tertile > 34.9	
		n = 283	n = 95	n = 94	n = 94	
ST segment elevation resolution post PCI, n (%)						
Complete, ≥70 %		128 (45)	56 (60)	42 (45)	30 (32)	
Incomplete, 30% to < 70%		114 (40)	32 (34)	36 (38)	46 (49)	0.002
None, ≤30%		40 (14)	6 (6)	16 (17)	18 (19)	
Initial blood results on admission						
C-reactive protein, (mg/L)	median (IQR)	4.0 (2.0 – 7.0)	3.0 (2.0 – 6.25)	4.0 (2.0 – 9.0)	3.0 (2.0 – 6.0)	0.150
	range	1.0 – 265.0	1.0 – 29.0	1.0 – 94.0	1.0 – 265.0	
Leucocyte cell count ($x10^9$L)		12.4 (3.6)	12.0 (3.1)	12.7 (3.6)	12.4 (3.9)	0.386
Neutrophil count ($x10^9$L)		9.6 (3.3)	9.2 (3.0)	9.8 (3.3)	9.9 (3.6)	0.293

Monocytes (x10^9L)	0.9 (0.4)	0.8 (0.3)	0.9 (0.3)	0.9 (0.5)	0.212
Platelet count (x10^6L)	246 (67)	254 (64)	248 (76)	236 (59)	0.195
IL-6, pg/ml	6.8 (4.7, 10.5)	5.9 (4.1, 8.2)	6.5 (4.8, 10.1)	9.5 (5.9, 18.6)	0.348
NT-proBNP, pg/mL	883 (323, 1621)	442 (248, 1084)	941 (518, 1661)	1253 (645, 1984)	<0.001
Medical therapy on discharge					
ACE-I or ARB	279 (99)	95 (100)	91 (97)	93 (99)	0.132
Beta-blocker	269 (95)	91 (96)	90 (96)	88 (94)	0.788

Footnote: IMR = index of microvascular resistance (mmHg x s, or Units); ACE-I or ARB = angiotensin converting enzyme inhibitor or angiotensin receptor blocker; LAD = Left anterior descending coronary artery; LCX = Left circumflex coronary artery; LM = left main coronary artery; RCA = right coronary artery; TIMI = Thrombolysis in Myocardial Infarction grade, PCI = percutaneous coronary intervention. Killip classification of heart failure after acute myocardial infarction: class I - no heart failure, class II - pulmonary rales or crepitations, a third heart sound, and elevated jugular venous pressure, class III - acute pulmonary edema, class IV - cardiogenic shock. * Data are reported as mean (SD), median (IQR), or N (%) as appropriate. P-values have been obtained from a one-way ANOVA or Fisher test. TIMI flow grades pre- and post-PCI were grouped 0/1 vs. 2/3 for this analysis. ‡ Diabetes mellitus was defined as a history of diet-controlled or treated diabetes. † Successfully

electrically cardioverted ventricular fibrillation at presentation or during emergency PCI procedure. ¥ Multivessel coronary artery disease was defined according to the number of stenoses of at least 50% of the reference vessel diameter, by visual assessment and whether or not there was left main stem involvement.

Table 2. Characteristics of 283 STEMI patients categorised according to tertile of CFR when measured acutely at the end of emergency PCI.

Characteristics*	All patients	Coronary flow reserve, CFR			P-value
		Lowest tertile<br>≤ 1.3	Mid-tertile<br>1.3 < CFR ≤ 1.8	Upper tertile<br>≥ 1.8	
	n = 283	n = 104	n = 91	n = 88	
Age, years	60 (12)	60 (11)	62 (12)	57 (11)	0.006
Male sex, n (%)	206 (73)	72 (69)	67 (74)	67 (76)	0.563
BMI, (kg/m^2)	29 (5)	29 (5)	28 (5)	29 (5)	0.585
Medical history					
Hypertension, n (%)	91 (32)	35 (34)	33 (36)	23 (26)	0.321
Current smoking, n (%)	175 (62)	66 (64)	52 (57)	57 (65)	0.532
Hypercholesterolaemia, n (%)	78 (28)	24 (23)	29 (32)	25 (28)	0.376
Diabetes mellitus‡, n (%)	30 (11)	17 (16)	9 (10)	4 (4)	0.028
Previous angina, n (%)	32 (11)	8 (8)	18 (20)	6 (7)	0.012
Previous myocardial infarction, n (%)	20 (7)	8 (8)	6 (7)	6 (7)	0.958

Previous PCI, n (%)		14 (5)	6 (6)	4 (4)	4 (4)	0.888
Presenting characteristics						
Heart rate, bpm		78 (17)	80 (17)	75 (16)	77 (18)	0.138
Systolic blood pressure, mmHg		136 (24)	135 (25)	133 (23)	139 (25)	0.204
Diastolic blood pressure, mmHg		79 (14)	79 (15)	77 (14)	82 (12)	0.124
Time from symptom onset to reperfusion, min		174 (120, 316)	170 (116, 307)	195 (121, 316)	166 (124, 316)	0.788
Ventricular fibrillation†, n (%)		19 (7)	5 (5)	7 (8)	7 (8)	0.636
Heart failure, Killip class at presentation, n (%)	I	201 (71%)	72 (69%)	60 (66%)	69 (78%)	
	II	62 (22%)	18 (17%)	27 (30%)	17 (19%)	0.008
	III / IV	20 (7)	14 (14)	4 (4)	2 (2)	
ECG						
ST segment elevation resolution post PCI, n (%)						
Complete, ≥70 %		128 (45)	18 (18)	10 (11)	12 (14)	
Incomplete, 30% to < 70%		114 (40)	45 (44)	40 (44)	29 (33)	0.247
None, ≤30%		40 (14)	40 (38)	41 (45)	47 (53)	

ST segment elevation resolution post PCI, n (%)						
< 50%		78 (28)	36 (35)	26 (29)	16 (18)	0.034
≥50%		204 (72)	67 (65)	65 (71)	72 (82)	
Reperfusion strategy, n (%)						
Primary PCI		262 (93)	96 (92)	83 (91)	83 (94)	
Rescue PCI (failed thrombolysis)		14 (5)	7 (7)	4 (4)	3 (3)	0.507
Successful thrombolysis		7 (2)	1 (1)	4 (4)	2 (2)	
Coronary angiography						
Number of diseased arteries¥, n (%)	1	158 (56)	59 (57)	52 (57)	47 (53)	
	2	83 (29)	34 (33)	20 (22)	29 (33)	0.337
	3	37 (13)	10 (10)	17 (19)	10 (11)	
	LM	5 (2)	1 (1)	2 (2)	2 (2)	
Culprit artery, n (%)	LAD	107 (38)	43 (41)	32 (35)	32 (36)	
	LCX	51 (18)	21 (20)	13 (14)	17 (19)	0.522
	RCA	125 (44)	40 (38)	46 (50)	39 (44)	
TIMI coronary flow grade pre-PCI, n (%)	0/1	204 (72)	88 (85)	57 (63)	59 (67)	

	2/3	79 (28)	16 (15)	34 (37)	29 (33)	0.001
TIMI coronary flow grade post-PCI, n (%)	0/1	3 (1)	0 (0)	1 (2)	1 (2)	
	2/3	281 (99)	103 (99)	90 (99)	88 (100)	1.000
TIMI frame count pre-PCI		29 (18, 44)	27 (16, 42)	27 (19, 37)	34 (24, 50)	0.244
TIMI frame count post-PCI		15 (10, 25)	17 (11, 25)	14 (9, 22)	15 (10, 28)	0.324
TIMI blush grade post-PCI	0/1	71 (26.4)	27 (32.1)	22 (22.9)	22 (24.7)	0.347
	2/3	198 (73.6)	57 (67.9)	74 (77.1)	67 (75.3)	
Culprit lesion, percentage residual stenosis		12.4 (5.5)	12.8 (5.6)	11.4 (5.5)	13.1 (5.4)	0.086
Coronary flow reserve		1.8 (0.9)	1.1 (0.2)	1.6 (0.1)	2.8 (0.0)	<0.001
Index of microvascular resistance		24 (15, 44)	28 (16, 49)	24 (15, 42)	21 (15, 33)	0.094
Fractional flow reserve		0.90 (0.10)	0.91 (0.08)	0.91 (0.09)	0.92 (0.08)	0.808
Medical therapy						
ACE-I or ARB		279 (99)	102 (98)	90 (99)	87 (99)	1.000
Beta-blocker		269 (95)	101 (97)	87 (96)	81 (92)	0.259
Treatment in the catheter laboratory						

Aspiration thrombectomy, n (%)		203 (71.7)	65 (73.9)	76 (73.1)	62 (68.1)	0.660
Glycoprotein IIbIIIa inhibitor		259 (91.5)	80 (90.9)	96 (92.3)	83 (91.2)	0.929
Initial blood results on admission						
C-reactive protein, (mg/L)	median (IQR)<br>range	4.0 (2.0 - 7.0)<br>1.0 - 265.0	3.0 (2.0 - 7.0)<br>1.0 - 265.0	4.0 (2.0 – 6.0)<br>1.0 - 94.0	4.0 (2.0 - 7.0)<br>1.0 - 92.0	0.740
Leucocyte cell count (x10^{9}L)		12.4 (3.6)	13.0 (3.7)	11.7 (3.1)	12.4 (3.8)	0.039
Neutrophil count (x10^{9}L)		9.6 (3.3)	10.4 (3.3)	9.0 (2.9)	9.5 (3.6)	0.011
Monocytes (x10^{9}L)		0.9 (0.4)	0.8 (0.4)	0.8 (0.3)	0.9 (0.3)	0.517
Platelet count (x10^{6}L)		246 (67)	250 (75)	246 (58)	240 (66)	0.591
IL-6, pg/ml		6.8 (4.7, 10.5)	7.7 (5.2, 10.8)	6.6 (4.6, 9.2)	5.8 (4.5, 8.6)	0.397
NT-proBNP, pg/mL		883 (323, 1621)	928 (359, 1469)	925 (399, 1726)	650 (293, 1412)	0.673

Footnote: CFR = coronary flow reserve (ratio with no units); ACE-I or ARB = angiotensin converting enzyme inhibitor or angiotensin receptor blocker; LAD = Left anterior descending coronary artery; LCX = Left circumflex coronary artery; LM = left main coronary artery; RCA = right coronary artery; TIMI = Thrombolysis in Myocardial Infarction grade, PCI = percutaneous coronary intervention. Killip classification of heart failure after acute myocardial infarction: class I - no heart failure, class II - pulmonary rales or crepitations, a third heart sound, and elevated jugular venous pressure, class III - acute pulmonary oedema, class IV - cardiogenic shock. * Data are reported as mean (SD), median (IQR), or N (%) as appropriate. P-values have been obtained from a one-way ANOVA or Fisher test. TIMI flow grades pre- and post-PCI were grouped 0/1 vs. 2/3 for this analysis. ‡ Diabetes mellitus was defined as a history of diet-controlled or treated diabetes. † Successfully electrically cardioverted ventricular fibrillation at presentation or during emergency PCI procedure. ¥ Multivessel coronary artery disease was defined

according to the number of stenoses of at least 50% of the reference vessel diameter, by visual assessment and whether or not there was left main stem involvement.

Table 3. Cardiac MRI findings at 2 days and 6 months post-reperfusion in 283 STEMI patients categorised according to tertile of CFR when measured acutely at the end of emergency PCI.

Characteristics*	All patients	Coronary flow reserve, CFR			P-value
		CFR ≤ 1.3	1.3 < CFR ≤ 1.8	CFR > 1.8	
	n = 283	n = 104	n = 91	n = 88	
CMR findings 2 days post-MI					
LV ejection fraction, %	55 (10)	54 (11)	55 (9)	56 (9)	0.708
LV end-diastolic volume, ml					
Men	160 (32)	162 (38)	158 (30)	161 (29)	0.754
Women	124 (25)	124 (17)	124 (31)	126 (27)	0.933
LV end-systolic volume, ml					
Men	74 (54, 92)	74 (48, 97)	73 (57, 86)	74 (55, 87)	0.528
Women	53 (41, 67)	54 (41, 67)	53 (40, 67)	48 (43, 65)	0.977
LV mass, g					
Men	141 (123, 160)	144 (128, 164)	141 (121, 153)	141 (126, 160)	0.490

Women	95 (85, 105)	94 (85, 102)	90 (85, 104)	101 (84, 116)	0.550
Oedema and infarct characteristics					
Area at risk, % LV mass	32 (12)	35 (12)	32 (11)	28 (11)	0.002
Infarct size, % LV mass	16 (7, 27)	19 (10, 32)	15 (6, 26)	13 (4, 22)	0.001
Late microvascular obstruction, n (%)	142 (50)	63 (61)	44 (48)	35 (40)	0.015
Late microvascular obstruction, % LV mass	0.1 (0.0, 3.5)	0.9 (0.0, 5.6)	0.0 (0.0, 2.8)	0.0 (0.0, 2.3)	0.012
CMR findings 6 months post-MI (n=267)					
LV ejection fraction at 6 months, %	63 (57, 70)	63 (54, 70)	64 (57, 69)	64 (59, 69)	0.634
LV end-diastolic volume at 6 months, ml					
Men	165 (136, 192)	176 (143, 204)	157 (131, 183)	165 (147, 191)	0.055
Women	121 (109, 136)	119 (109, 131)	136 (113, 149)	120 (102, 133)	0.176
Change in LV end-diastolic volume at 6 months from baseline, ml					
Men	6.3 (-7.9, 20.6)	13.0 (-.18, 32.6)	-3.2 (-17.5, 12.2)	7.3 (-6.2, 17.4)	0.002
Women	0.9 (-11.4, 9.9)	0.4 (-11.2, 6.8)	5.1 (-7.7, 16.3)	-2.2 (-12.4, 8.6)	0.465

Footnote: Abbreviations: LV = left ventricle, T1 = myocardial longitudinal relaxation time. Area-at-risk was measured with T2-mapping. Data are given as n (%) or mean (SD). P-values were obtained from one-way ANOVA, Kruskal-Wallis test, or a Fisher test. * Data are reported as mean (SD), median (IQR), or n (%) as appropriate.

Table 4. The diagnostic accuracy for IMR and CFR at various cut-offs for microvascular obstruction and myocardial haemorrhage.

Test	Outcome				Sensitivity	Specificity	Positive predictive value (PPV)	Negative predictive value (NPV)	Diagnostic accuracy*
IMR >40	Myocardial hemorrhage		Yes	No	0.43 (0.32, 0.54)	0.78 (0.70, 0.85)	0.58 (0.46, 0.71)	0.66 (0.57, 0.73)	0.63 (0.56, 0.70)
		>40	38	27					
		≤40	51	97					
IMR >40	Microvascular obstruction		Yes	No	0.38 (0.30, 0.47)	0.82 (0.75, 0.88)	0.68 (0.57, 0.78)	0.67 (0.50, 0.64)	0.60 (0.54, 0.66)
		>40	54	25					
		≤40	88	116					
IMR >27	Myocardial hemorrhage		Yes	No	0.66 (0.55, 0.76)	0.67 (0.60, 0.77)	0.61 (0.50, 0.71)	0.74 (0.65, 0.82)	0.68 (0.61, 0.74)
		>27	59	38					
		≤27	30	86					
IMR >27	Microvascular obstruction		Yes	No	0.58 (0.49, 0.66)	0.72 (0.63, 0.79)	0.67 (0.58, 0.75)	0.63 (0.55, 0.70)	0.65 (0.59, 0.70)
		>27	82	40					
		≤27	60	101					

CFR <1.5	Myocardial hemorrhage		Yes	No	0.53 (0.42, 0.63)	0.65 (0.55, 0.73)	0.52 (0.41, 0.62)	0.66 (0.56, 0.74)	0.60 (0.53, 0.66)
		<1.5	47	44					
		≥1.5	42	80					
CFR <1.5	Microvascular obstruction		Yes	No	0.48 (0.39, 0.56)	0.63 (0.55, 0.71)	0.57 (0.47, 0.66)	0.55 (0.47, 0.62)	0.55 (0.49, 0.61)
		<1.5	68	52					
		≥1.5	74	89					
CFR <2.0	Myocardial hemorrhage		Yes	No	0.80 (0.70, 0.88)	0.34 (0.26, 0.43)	0.46 (0.38, 0.55)	0.70 (0.57, 0.71)	0.53 (0.46, 0.60)
		<1.5	71	82					
		≥1.5	18	42					
CFR <2.0	Microvascular obstruction		Yes	No	0.79 (0.71, 0.85)	0.34 (0.26, 0.42)	0.55 (0.48, 0.62)	0.62 (0.50, 0.72)	0.57 (0.51, 0.62)
		<1.5	112	93					
		≥1.5	30	48					

Denominator, n = 283 with both IMR and CFR available. * Diagnostic accuracy is defined as the proportion of all tests that give a correct result.

Table 5. Multivariable associations between clinical characteristics at presentation, including CFR (for a 0.5 unit difference in CFR) at the end of emergency PCI, and the occurrence of microvascular obstruction two days later (n=200) in patients with acute STEMI.*

Binary logistic regression	Odds ratio (95% CI)	p value
Coronary flow reserve (0.5 unit difference in CFR)	*0.02 (0.00, 0.13)*	*<0.001*
Age (years)	1.00 (0.97, 1.03)	0.979
Male gender	*2.19 (1.14, 4.18)*	*0.018*
BMI	0.99 (0.93, 1.05)	0.804
Previous MI	0.59 (0.13, 2.63)	0.487
Diabetes mellitus	0.93 (0.37, 2.36)	0.881
Previous PCI	6.18 (0.92, 41.30)	0.060
Smoker	1.61 (0.84, 3.10)	0.154
Hypertension	1.27 (0.67, 2.43)	0.466
Hypercholesterolaemia	1.03 (0.51, 2.10)	0.935
Previous angina	0.89 (0.35, 2.31)	0.818

Heart rate (bpm)	0.99 (0.97, 1.01)	0.424
SBP per 10 mmHg	1.03 (0.92, 1.17)	0.588
Symptom to reperfusion time per 10 minutes	*1.02 (1.01, 1.04)*	*0.007*
TIMI blush grade post-PCI 2/3	0.86 (0.45, 1.63)	0.643
Culprit lesion, percentage residual stenosis	1.00 (0.94, 1.05)	0.860
No-resolution of ST-elevation	2.21 (0.95, 5.13)	0.066
Harrel's C-statistic:	0.731	

*TIMI frame count was excluded due to numerical instability. The Harrel's C-statistic reflects the predictive power of the multivariable model.

Table 6. Multivariable associations between clinical characteristics at presentation, including CFR (for a 5 unit difference in CFR) at the end of emergency PCI, and the occurrence of myocardial haemorrhage two days later (n=200) in patients with acute STEMI.*

Binary logistic regression	Odds ratio (95% CI)	p value
CFR (0.5 unit difference in CFR)	*0.02 (0.00, 0.20)*	*0.001*
Age (years)	1.02 (0.99, 1.06)	0.239
Male gender	*3.13 (1.36, 7.19)*	*0.007*
BMI	1.00 (0.92, 1.08)	0.977
Previous MI	0.76 (0.14, 4.05)	0.751
Diabetes mellitus	1.17 (0.40, 3.42)	0.767
Previous PCI	4.20 (0.56, 31.60)	0.164
Smoker	*3.32 (1.45, 7.58)*	*0.004*
Hypertension	1.19 (0.55, 2.58)	0.656
Hypercholesterolaemia	1.82 (0.73, 4.52)	0.195
Previous angina	0.83 (0.27, 2.58)	0.749

Heart rate (bpm)	1.00 (0.98, 1.02)	0.910
SBP per 10 mmHg	0.99 (0.86, 1.15)	0.932
Symptom to reperfusion time per 10 minutes	1.01 (1.00, 1.02)	0.192
TIMI blush grade post-PCI 2/3	0.79 (0.38, 1.64)	0.529
Culprit lesion, percentage residual stenosis	1.03 (0.96, 1.09)	0.429
No-resolution of ST-elevation	2.10 (0.82, 5.35)	0.120
Harrel's C-statistic:	0.745	

*TIMI frame count was excluded due to numerical instability. The Harrel's C-statistic reflects the predictive power of the multivariable model.

Table 7. Clinical and angiographic characteristics of 121 STEMI patients who had IMR measured at the end of emergency PCI and a blood test obtained 1 – 2 day later during the index hospitalisation for NT-proBNP and Il-6.

Characteristics*	All patients	Index of microvascular resistance, IMR			P-value
		Lowest tertile<br>≤ 17	Mid-tertile<br>17 - ≤ 34.9	Upper tertile<br>> 34.9	
	n = 121	n = 40	n = 46	n = 35	
Age, years	59.1 (11.6)	58.7 (10.9)	58.0 (11.9)	68.9 (12.0)	0.532
Male sex, n (%)	95 (78.5)	32 (80.0)	34 (73.9)	29 (82.9)	0.593
BMI, (kg/m^2)	28.7 (4.3)	28.6 (4.6)	29.3 (4.3)	28.2 (4.1)	0.517
Medical history					
Hypertension, n (%)	35 (28.9)	15 (37.5)	9 (19.6)	11 (31.4)	0.178
Current smoking, n (%)	71 (58.7)	25 (62.5)	29 (63.0)	17 (48.6)	0.386
Hypercholesterolemia, n (%)	30 (24.8)	10 (25.0)	9 (19.6)	11 (31.4)	0.483
Diabetes mellitus‡, n (%)	11 (9.1)	5 (12.5)	1 (2.2)	5 (14.3)	0.084
Previous angina, n (%)	21 (17.4)	3 (7.5)	11 (23.9)	7 (20.0)	0.101
Previous myocardial infarction, n (%)	7 (5.8)	2 (5.0)	3 (6.5)	2 (5.7)	1.000

Previous PCI, n (%)		6 (5.0)	0 (0)	2 (4.3)	4 (8.9)	0.062
Presenting characteristics						
Heart rate, bpm		78.0 (16.1)	80.8 (15.8)	73.4 (15.3)	81.0 (16.6)	0.047
Systolic blood pressure, mmHg		135.9 (25.5)	139.4 (27.3)	131.3 (24.3)	138.1 (24.7)	0.294
Diastolic blood pressure, mmHg		79.1 (13.9)	76.5 (14.6)	78.6 (14.7)	82.7 (11.7)	0.159
Time from symptom onset to reperfusion, min		171 (121, 307)	158 (116, 254)	174 (131, 299)	176 (130, 510)	0.396
Ventricular fibrillation†, n (%)		5 (4.3)	1 (2.5)	3 (6.5)	1 (2.9)	0.627
Heart failure, Killip class at presentation, n (%)	I	92 (76.0)	34 (85)	34 (73.9)	24 (69.6)	0.322
	II	24 (19.8)	6 (15)	10 (21.7)	8 (22.9)	
	III / IV	5 (4.1)	0 (0)	2 (4.3)	3 (8.6)	
ECG						
ST segment elevation resolution post PCI, n (%)						
Complete, ≥70 %		58 (47.9)	26 (65.0)	21 (45.7)	11 (31.4)	0.027
Incomplete, 30% to < 70%		50 (41.3)	13 (32.5)	18 (39.1)	19 (54.3)	
None, ≤30%		13 (10.7)	1 (2.5)	7 (15.2)	5 (31.4)	
ST segment elevation resolution post PCI, n (%)						
< 50%		32 (26)	1 (2)	14 (30)	17 (49)	<0.001

≥50%		89 (74)	39 (98)	32 (70)	18 (51)	
Reperfusion strategy, n (%)						
Primary PCI		114 (94.2)	39 (97.5)	41 (89.1)	34 (97.1)	0.526
Rescue PCI (failed thrombolysis)		5 (4.1)	1 (2.5)	3 (6.5)	1 (2.9)	
Successful thrombolysis (convalescent STEMI)		2 (1.7)	0 (0)	2 (4.3)	0 (0)	
Coronary angiography						
Number of diseased arteries¥, n (%)	1	74 (61.2)	25 (62.5)	27 (58.7)	22 (62.9)	0.781
	2	34 (28.1)	10 (25.0)	15 (32.6)	9 (25.7)	
	3	10 (8.3)	4 (10.0)	2 (4.3)	4 (11.4)	
	LM	3 (2.5)	1 (2.5)	2 (4.3)	0 (0)	
Culprit artery, n (%)	LAD	45 (37.2)	14 (35.0)	19 (41.3)	12 (34.3)	0.826
	LCX	25 (20.7)	9 (22.5)	7 (15.2)	9 (25.7)	
	RCA	51 (42.1)	17 (42.5)	20 (43.5)	14 (40.0)	
TIMI coronary flow grade pre-PCI, n (%)	0/1	90 (74.4)	25 (62.5)	34 (73.9)	31 (88.6)	
	2/3	31 (25.6)	15 (37.5)	12 (26.1)	4 (11.4)	0.034
TIMI coronary flow grade post-PCI, n (%)	0/1	0 (0)	0 (0)	0 (0)	0 (0)	1.000
	2/3	121 (100)	40 (100)	46 (100)	35 (100)	

TIMI frame count pre-PCI		39 (28, 51)	41 (34, 50)	30 (21, 48)	50 (38, 62)	0.366
TIMI frame count post-PCI		14 (10, 24)	14 (8, 22)	14 (10, 20)	22 (14, 30)	0.001
TIMI blush grade post-PCI	0/1	27 (23.9)	3 (7.9)	9 (21.4)	15 (45.5)	<0.001
	2/3	86 (76.1)	35 (92.1)	33 (78.6)	18 (54.5)	
Culprit lesion, percentage residual stenosis		12.4 (5.9)	11.6 (6.2)	13.4 (5.7)	12.1 (5.7)	0.346
Coronary flow reserve		1.5 (1.1, 1.9)	1.4 (1.1, 1.8)	1.8 (1.4, 2.4)	1.2 (1.1, 1.6)	<0.001
Index of microvascular resistance		23.5 (16.0, 37.0)	13.2 (10.0, 16.0)	24.0 (20.8, 28.8)	51.0 (40.5, 76.9)	<0.001
Fractional flow reserve		0.93 (0.87, 0.97)	0.89 (0.84, 0.93)	0.94 (0.87, 0.97)	0.95 (0.91, 0.99)	0.003
Aspiration thrombectomy, n (%)		90 (74.4)	27 (67.5)	37 (80.4)	26 (74.3)	0.426
Glycoprotein IIbIIIa inhibitor		112 (92.6)	38 (95.0)	41 (89.1)	33 (94.3)	0.681

Footnote: IMR = index of microvascular resistance (mmHg x s, or Units); ACE-I or ARB = angiotensin converting enzyme inhibitor or angiotensin receptor blocker; LAD = Left anterior descending coronary artery; LCX = Left circumflex coronary artery; LM = left main coronary artery; RCA = right coronary artery; TIMI = Thrombolysis in Myocardial Infarction grade, PCI = percutaneous coronary intervention. Killip classification of heart failure after acute myocardial infarction: class I - no heart failure, class II - pulmonary rales or crepitations, a third heart sound, and elevated jugular venous pressure, class III - acute pulmonary edema, class IV - cardiogenic shock. * Data are reported as mean (SD), median (IQR), or N (%) as appropriate. P-values have been obtained from a one-way ANOVA, Kruskal-Wallis test or Fisher test. TIMI flow grades pre- and post-PCI were grouped 0/1 vs. 2/3 for this analysis. ‡ Diabetes mellitus was defined as a history of diet-controlled or treated diabetes. † Successfully electrically cardioverted ventricular fibrillation at presentation or during emergency PCI procedure. ¥ Multivessel coronary artery disease was defined according to the number of stenoses of at least 50% of the reference vessel diameter, by visual assessment and whether or not there was left main stem involvement.

Table 8. Cardiac MRI findings in 121 STEMI patients categorized according to tertile of IMR measured at the end of emergency PCI and a blood test obtained 1 – 2 day later during the index hospitalisation for NT-proBNP and Il-6.

Characteristics	All patients<br>n = 121	IMR ≤ 17<br>n = 40	17 < IMR ≤ 34.9<br>n = 46	IMR > 34.9<br>n = 35	P-value
MRI findings 2 days post-MI					
LV ejection fraction, %	55.7 (9.6)	59.5 (8.6)	54.9 (10.2)	52.4 (8.5)	0.004
LV end-diastolic volume, ml					
Men	156.7 (32.9)	156.5 (40.5)	154.5 (29.5)	159.6 (27.9)	0.832
Women	127.8 (20.1)	125.1 (17.1)	133.8 (22.0)	119.1 (22.2)	0.346
LV end-systolic volume, ml					
Men	69.5 (49.9, 86.7)	61.3 (49.8, 79.6)	68.7 (47.7, 89.7)	72.6 (62.6, 94.9)	0.315
Women	56.3 (42.8, 68.3)	41.7 (39.5, 60.8)	63.3 (45.2, 75.3)	56.7 (53.5, 61.7)	0.245
Edema and infarct characteristics					
Extent of myocardial edema, % LV mass	30.5 (11.2)	26.4 (8.3)	30.2 (11.6)	35.6 (11.7)	0.001
Infarct size, % LV mass	16.8 (12.5)	11.4 (8.5)	16.8 (13.6)	23.0 (12.12)	<0.001
Late microvascular obstruction, n (%)	58 (47.9)	12 (30.0)	22 (47.8)	24 (68.6)	0.004

Late microvascular obstruction, % LV mass (n=58)	2.99 (1.61, 7.18)	1.61 (0.77, 3.49)	2.30 (1.45, 6.64)	5.57 (2.07, 7.86)	0.063
MRI findings 6 months post-MI (n=259)					
LV ejection fraction at 6 months, %	62.9 (8.5)	65.7 (7.1)	63.0 (9.0)	59.3 (8.3)	0.008
Change in LV end-diastolic volume at 6 months from baseline, ml					
Men	3.8 (25.7)	-3.2 (30.0)	5.8 (24.0)	9.3 (21.2)	0.175
Women	-2.1 (23.7)	-4.1 (17.9)	3.7 (26.3)	-8.0 (28.3)	0.636

Footnote: Abbreviations: LV = left ventricle, T1 = myocardial longitudinal relaxation time. Area-at-risk was measured with T2-mapping. Data are given as n (%) or mean (SD). P-values were obtained from one-way ANOVA, Kruskal-Wallis test, or a Fisher test. * Data are reported as mean (SD), median (IQR), or n (%) as appropriate. *MRI data were not available in 2 patients.

Table 9. Multivariable association between IMR at the end of emergency PCI, and the change in left ventricular end-diastolic volume at 6 months from baseline in 245 patients following acute STEMI, after adjustment for baseline predictors.

Multiple stepwise regression	Coefficient (95% CI)	p value
IMR (1.0 unit difference)	0.13 (0.01, 0.24)	0.036
LV end-diastolic volume, ml	-0.17 (-0.28, -0.05)	0.005
Male gender	12.98 (4.77, 21.19)	0.002
Hypercholesterolemia	-8.66 (-16.58, -0.75)	0.032
Killip class 3 or 4 heart failure	30.41 (15.95, 44.86)	<0.001
Sustained ventricular arrhythmia	16.03 (2.86, 29.20)	0.017

Multivariable stepwise regression analysis, the coefficient and 95% confidence intervals indicate the magnitude and direction of the difference in LV end-diastolic volume (ml) at follow-up from baseline LV end-diastolic volume for the patient characteristic (binary or continuous). For example, on average, LV end-diastolic volume (ml) at follow-up is 0.13 (0.01, 0.24) higher for each 1 unit increase in IMR at baseline. The other variables that were included in this model were age (p=0.190), BMI (0.201), previous myocardial infarction (p=0.976), diabetes mellitus (p=0.097), previous PCI (p=0.825), cigarette smoker (p=0.641), hypertension (p=0.331), previous angina (p=0.705), heart rate (p=0.643), systolic blood pressure at initial angiography (p=0.302), Killip class 2 (p=0.671), symptom to reperfusion time (p=0.339), TIMI flow grade at initial angiography (p=0.217), no ST-segment resolution (<30%)(p=0.107), or partial ST-segment resolution (30-70%) (p=0.765).

Table 10. Multivariable association IMR and CFR at the end of emergency PCI and the changes in LV ejection fraction at 6months from baseline in 239 STEMI survivors with adjustment for baseline predictors.

Multiple stepwise regression	Coefficient (95% CI)	p value
LVEF at baseline	-0.44 (-0.56, -0.32)	<0.001
IMR (for a 5 unit difference in IMR)	-0.24 (-0.40, -0.07)	0.006
Male sex	-1.92 (-3.97, -0.13)	0.066
Previous myocardial infarction	-6.04 (-10.61, -1.46)	0.010
Multiple stepwise regression	coefficient (95% CI)	p value
LVEF at baseline	-0.42 (-0.53, -0.30)	<0.001
CFR (for a 0.2 unit difference in CFR)	0.21 (0.01, 0.41)	0.043
Male sex	-2.17 (-4.23, -0.10)	0.040
Previous myocardial infarction	-5.70 (-10.31, -1.08)	0.016

Multivariable stepwise regression analysis, the coefficient and 95% confidence intervals indicate the magnitude and direction of the difference in LV ejection fraction (LVEF, %) at follow-up from baseline LVEF for the patient characteristic (binary or continuous). For example, on average, LVEF (%) at follow-up is 0.24 (0.40, 0.07) % points lower for each 5 unit increase in IMR at baseline. The other variables that were included in these models were age, male sex, BMI, diabetes mellitus, previous PCI, cigarette smoker, hypertension, previous angina, heart rate, systolic

blood pressure at initial angiography, Killip class, symptom to reperfusion time, TIMI flow grade at initial angiography, no ST-segment resolution (<30%), or partial ST-segment resolution (30-70%) .

Figure 1.

(a) CFR at baseline and LV ejection fraction 6 months later.

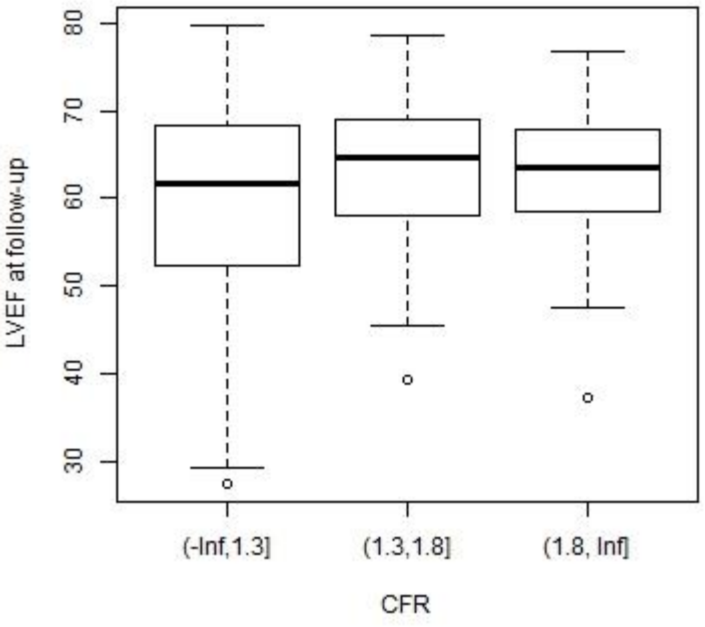

(b) IMR at baseline and LV ejection fraction 7 months later.

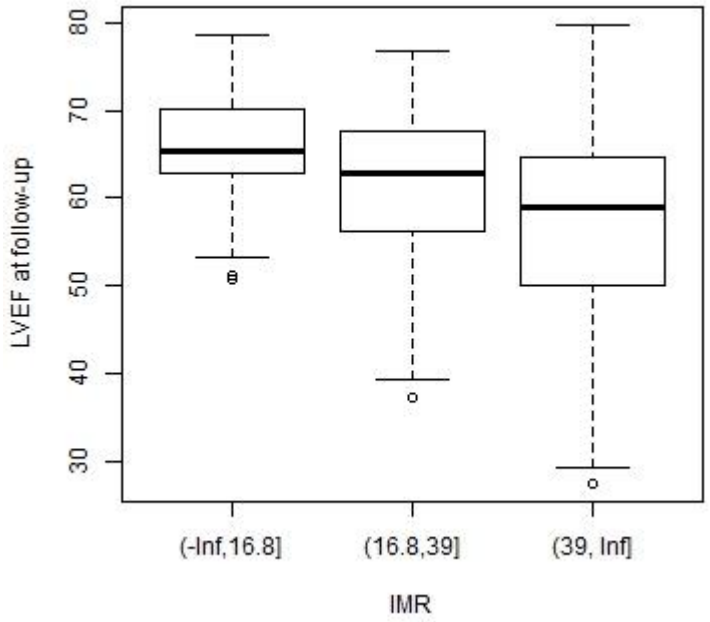

Figure 2. Bland-Altman plot for inter-observer agreement of infarct core T2* values.

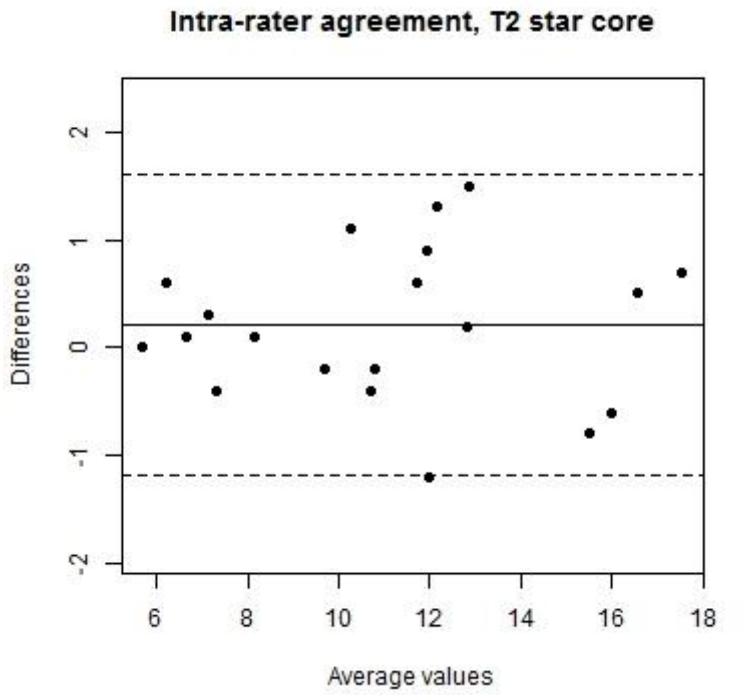

Supplement: Supplemental data [file jciinsight-1-85768-s001.pdf]
